# miRNome expression analysis in canine diffuse large B-cell lymphoma

**DOI:** 10.3389/fonc.2023.1238613

**Published:** 2023-08-30

**Authors:** Nelly O. Elshafie, Michael Gribskov, Nathanael I. Lichti, Ekramy. E. Sayedahmed, Michael O. Childress, Andrea P. dos Santos

**Affiliations:** ^1^ Department of Comparative Pathobiology, Purdue University, West Lafayette, IN, United States; ^2^ Department of Biological Sciences, Purdue University, West Lafayette, IN, United States; ^3^ Bindley Bioscience Center, Purdue University, West Lafayette, IN, United States; ^4^ Department of Veterinary Clinical Sciences, Purdue University, West Lafayette, IN, United States

**Keywords:** diffuse large B-cell lymphoma, multicentric, miRNAs, cancer biomarkers, miRNome, sRNA sequencing, canine DLBCL

## Abstract

**Introduction:**

Lymphoma is a common canine cancer with translational relevance to human disease. Diffuse large B-cell lymphoma (DLBCL) is the most frequent subtype, contributing to almost fifty percent of clinically recognized lymphoma cases. Identifying new biomarkers capable of early diagnosis and monitoring DLBCL is crucial for enhancing remission rates. This research seeks to advance our knowledge of the molecular biology of DLBCL by analyzing the expression of microRNAs, which regulate gene expression by negatively impacting gene expression via targeted RNA degradation or translational repression. The stability and accessibility of microRNAs make them appropriate biomarkers for the diagnosis, prognosis, and monitoring of diseases.

**Methods:**

We extracted and sequenced microRNAs from ten fresh-frozen lymph node tissue samples (six DLBCL and four non-neoplastic).

**Results:**

Small RNA sequencing data analysis revealed 35 differently expressed miRNAs (DEMs) compared to controls. RT-qPCR confirmed that 23/35 DEMs in DLBCL were significantly upregulated (n = 14) or downregulated (n = 9). Statistical significance was determined by comparing each miRNA's average expression fold-change (2-Cq) between the DLCBL and healthy groups by applying the unpaired parametric Welch's 2-sample t-test and false discovery rate (FDR). The predicted target genes of the DEMs were mainly enriched in the PI3K-Akt-MAPK pathway.

**Discussion:**

Our data point to the potential value of miRNA signatures as diagnostic biomarkers and serve as a guideline for subsequent experimental studies to determine the targets and functions of these altered miRNAs in canine DLBCL.

## 1 Introduction

Lymphomas are a complex, heterogeneous group of hematopoietic malignancies with similarities between dogs and humans (1), accounting for 7- 24% of all cancers in dogs and approximately 83% of all hematopoietic cancers (2). Canine lymphomas microscopically resemble some forms of non-Hodgkin lymphomas in humans and respond similarly to standard treatments (3). Diffuse large B-cell lymphoma (DLBCL) is humans’ most frequent aggressive lymphoma subtype, representing almost 30% of NHL cases (4). DLBCL is also the most reported subtype of lymphoma in dogs, followed by peripheral T-cell lymphoma not otherwise specified, nodal T-zone lymphoma, T-lymphoblastic lymphoma, and marginal zone lymphoma (5).

Several approaches are used to treat DLBCL, depending on the cancer stage and response to treatment. CHOP-based chemotherapy (cyclophosphamide, doxorubicin, vincristine, prednisone) (6, 7) is the standard treatment for DLBCL, with overall survival times varying from 10 to 14 months (7). CHOP therapy is not curative; but diminishes the cancer burden, resulting in partial to complete remission (8). Nevertheless, lymphoma patients eventually experience cancer relapse, resulting from the inability of chemotherapy to eradicate the subclinical disease. Earlier detection of DLCBL may improve the chances of therapeutic success. Thus, there is a continuous search for additional biomarkers capable of early detecting and monitoring responses to treatment in dogs with DLBCL.

The diagnosis of DLBCL relies on histologic evaluation of a tissue biopsy in conjunction with immunohistochemical (IHC) or flow cytometry analysis (9). This workflow is sufficient to confirm the diagnosis; however, it may be challenging to differentiate reactive lymph nodes from DLCBL in some cases (9). PCR for antigen receptor rearrangements (PARR) assays to determine clonality assays aid in a more accurate diagnosis (10); however, the workflow becomes laborious and expensive. A diagnostic test capable of detecting DLBCL and identifying prognostically relevant patient subgroups would substantially simplify the ability to diagnose and treat DLBCL correctly.

This study aims to broaden our understanding of the molecular biology of canine DLBCL by investigating the role of gene expression regulators called microRNAs (miRNA) as possible diagnostic biomarkers in DLBCL. MicroRNAs are small non-coding RNAs (11) that negatively modulate gene expression (12–14); they are incorporated into the RNA silencing complex and, in the case of perfect complementarity at the 3’ untranslated region with the mRNA target, induce targeted mRNA degradation (15). In the case of imperfect complementarity, miRNAs may block gene expression at the translational level (13, 16–19). One miRNA can target various genes, including transcriptional activators and repressors, thereby regulating physiological and pathological processes (18, 20). Mature miRNAs are abundant in the cell cytoplasm and circulate extracellularly in bodily fluids such as plasma and serum (21, 22) packaged in lipoprotein complexes, which protect their degradation by ribonucleases (23, 24), permitting their uptake by distant cells, where they also contribute to cell-to-cell communication (17, 25).

Aberrant expression of miRNA has been seen in numerous diseases, including cancers (17, 26, 27). Most miRNA genes occupy mutable sites within the genome. These regions are also enriched in other cancer-related genes (26, 28); consequently, miRNAs have drawn considerable attention as potential cancer biomarkers. MicroRNAs can be used as diagnostic and prognostic biomarkers to detect disease subtypes (29, 30). They can also be measured sequentially to evaluate the evolution in disease status over time (30–33) or the response to treatment (34), as they play crucial roles in regulating cancer progression.

Compared to healthy dogs, the miRNA profile of the canine DLBCL-affected dogs is not thoroughly studied. This study investigates the differentially expressed miRNAs in canine DLBCL using fresh frozen lymph node samples from six DLBCL dog patients and four normal healthy dogs. This study enhances our understanding of the canine DLBCL miRNAs’ signature profile, which could be a valuable tool in disease detection and progression in dogs. This study also improves our understanding of the disease mechanism, molecular pathways, biomarkers discovery, dysregulated miRNAs, and personalized medicine.

## 2 Materials and methods

### 2.1 Animal samples

Ten fresh-frozen lymph node biopsy specimens were used in this study. Six of these samples were affected by DLBCL, and 4 were histologically normal lymph nodes. Dogs with DLBCL were presented to the Purdue University Veterinary Teaching Hospital (PUVTH), West Lafayette, IN, from 2013 to 2014 for cancer diagnosis, staging, and treatment. All samples from dogs with DLCBL were collected by surgical biopsy of an affected peripheral lymph node (incisional wedge biopsy or surgical extirpation of an entire lymph node) while the dogs were under general anesthesia. The diagnosis of DLBCL was made by integrating histopathology and immunohistochemistry results confirming the expression of CD79a and/or Pax-5 and lack of expression of CD3 by the neoplastic cells. The controls used in the study were four fresh-frozen, non-neoplastic lymph nodes collected from purpose-bred research dogs from a commercial vendor (Covance Research Products, Inc.). with no substantial clinical, hematologic, or biochemical abnormalities. These dogs were humanely euthanatized in academic surgical laboratory courses taught to veterinary students at the Purdue College of Veterinary Medicine. Animal medical history and test results of DLBCL cases are provided (**Supplementary Table 1A**). The demographic data of healthy cases are demonstrated (**Supplementary Table 1B**). The samples were evaluated and classified by a board-certified pathologist according to the WHO criteria of 2008. All the samples for this project were collected with informed consent from the dogs’ owners, and procedures for sample collection were approved by the Purdue Animal Care and Use Committee (protocols #1708001607 and #1111000308). All lymph node samples were frozen in liquid nitrogen immediately after harvesting and stored at -80°C until total miRNA extraction.

### 2.2 RNA extraction

RNA extraction was performed using the Monarch^®^ Total RNA Miniprep Kit (NEB, Ipswich, MA, USA). Briefly, the fresh-frozen samples were quickly thawed in an equal volume of the Monarch^®^ DNA/RNA Protection Reagent and homogenized using a vortex. Then, 10 µL of Proteinase K was added to the mixture. After a brief vortex, the samples were incubated at room temperature for 30 minutes; then, an equal amount of isopropanol was added, followed by a quick vortex. The mixture was passed through an RNA purification column and washed twice using a 500 µL RNA Wash Buffer. Subsequently, DNase I treatment was used to remove residual gDNA. A 500 µL RNA Priming Buffer was added for RNA binding, followed by a few washing steps. Afterward, the total RNA was eluted with 100 µL nuclease-free water, and the RNA samples were stored at -80°C. Before sequencing, the quantity and quality of total RNA were assessed by UV^®^ spectrophotometry (NanoDrop, ThermoFisher Scientific, Waltham, MA, USA). The quantification of small RNAs was assisted by a spectrofluorometer (Qubit 4 Fluorometer, ThermoFisher Scientific, Waltham, MA, USA) using Invitrogen™ Qubit™ microRNA Assay Kits (ThermoFisher Scientific, Waltham, MA, USA) as demonstrated (**Supplementary Table 2**).

### 2.3 Library preparation and sequencing

#### 2.3.1 RNA quantification and qualification

The extracted total RNA from each sample was sequenced (Novogene Co., Ltd., Beijing, China). RNA degradation and contamination were examined on 1% agarose gels. RNA purity was tested utilizing the Nanophotometer^®^ UV spectrophotometer (IMPLEN, Westlake Village, CA, USA). RNA integrity and quantification were measured utilizing the RNA Nano 6000 Assay Kit of the Agilent Bioanalyzer 2100 system (Agilent Technologies, Santa Clara, CA, USA).

#### 2.3.2 Library construction and sequencing for sRNA-seq

The small RNA library construction used 3 μg of total RNA per sample. Following the manufacturer’s recommendations, sequencing libraries were produced using NEBNext^®^ Multiplex Small RNA Library Prep Set (NEB, Ipswich, MA, USA) for Illumina^®^, and index codes were added to allow attribution of sequences to samples. First-strand cDNA was performed using M-MuLV Reverse Transcriptase (RNase H–), PCR amplification using LongAmp Taq 2X Master Mix, SR Primer for Illumina, and index (X) primer. The purified PCR products were examined on an 8% polyacrylamide gel (100V, 80 min). DNA fragments equivalent to 140~160 bp (the length of small non-coding RNA plus the 3’ and 5’ adaptors) were excised and suspended in an eight μL elution buffer. Lastly, library quality was evaluated using DNA High Sensitivity Chips on the Agilent Bioanalyzer 2100 system (Agilent Technologies, Santa Clara, CA, USA).

### 2.4 Data analysis

#### 2.4.1 Quality control

Customized Perl (https://www.perl.org/get.html) and Python (https://www.python.org/downloads/) scripts were used to analyze the raw fastq files. The fraction of reads with < 1% (Q20) and 0.1% (Q30) error, GC-content, were calculated after cleaning up reads with ploy-N, 5’ adapter contaminants without 3’ adapter, or the insert tag, in addition to reads containing poly A, T, G, C, or having low-quality as shown (**Supplementary Tables 3**, **4**).

#### 2.4.2 Reads mapping to the reference sequence

The small RNA sequences were mapped to the reference sequence using Bowtie 0.12.9 (35), as shown (**Supplementary Table 5**). For known miRNAs, miRBase20.0 and modified software miRDeep2_0_0_5 were used as a reference (36). Small RNA tags were mapped to RepeatMasker open-4.0.3 and Rfam (37) databases to remove tags originating from repetitive sequences, rRNA, tRNA, snRNA, and snoRNA. The software package miRDeep2 (36) and the characteristics of the hairpin structure of miRNA precursor were integrated to predict potential novel miRNAs.

#### 2.4.3 Differentially expressed miRNAs

Expression levels were assessed as transcripts per million (TPM) using the following criteria (38): (1) Normalization formula: Normalized expression = mapped read count/total reads*1000000. (2) Differential expression analysis of the two groups (DLBCL vs. healthy) is displayed (**Supplementary Table 6**) and was achieved using the DESeq2 v1.12.0 and R package (1.8.3) (39). The False Discovery Rate (FDR) rate was controlled by adjusting the P-values using the Benjamini & Hochberg (BH) method implemented in DESeq2. Adjusted P-values of <0.05 and the fold change of > 2 were used as the threshold for significantly differential expression. Predicting the target gene of miRNA was performed by miRanda3.3a (40).

#### 2.4.4 GO and KEGG enrichment

Gene Ontology (GO) enrichment analysis was used to identify conserved biological processes, molecular functions, and cellular compartments related to the predicted gene targets of the differentially expressed miRNAs (DEMs). The GOseq R package (Release 2.12) based on Wallenius non-central hypergeometric distribution (41) and adjusted *P-values* BH of <0.05, which adjusts for gene length bias, was implemented for enrichment analysis.

Over-represented pathways were identified based on the predicted target genes of the DEMs and pathways defined in the Kyoto Encyclopedia of Genes and Genomes (KEGG) (41). KOBAS V3.0 software was used to test the statistical enrichment of the target gene candidates in KEGG pathways (42). To graphically visualize the enrichment pathway, ShinyGO 0.75 (43) was used to annotate pathways from multiple resources: GO, KEGG, Reactome (44, 45), PANTHER (46), and WikiPathways (47). The KEGG diagram was retrieved using KEGG and path view (48, 49).

### 2.5 Validation of the small RNA sequencing results through quantitative RT-PCR

Following the manufacturer’s protocol, the first-strand cDNA synthesis was performed using the miRCURY LNA RT Kit (QIAGEN, Germantown, MD, USA). UniSp6 RNA spike-in, the reverse transcription control, was diluted and added to the mix as instructed. Total RNA was diluted to 50 ng/μL in RNase−free water. The total volume of the reaction mixture (10 μL) was kept on ice to avoid RNA degradation. The reaction mixture consisted of 2 μL of 5x miRCURY SYBR^®^ Green Reaction Buffer, 4.5 μl RNase-free Water, 1 μL of 10x miRCURY RT Enzyme Mix, 2 μL of the diluted RNA, and 0.5 μL of RNA synthetic spike−in (UniSp6). Reverse-transcription thermal cycling was performed as instructed: incubation for 1 h at 42°C, followed by reverse transcriptase enzyme inactivation for 5 min at 95°C, then immediate cooling to 4°C. The synthesized cDNA was diluted, and quantitative RT-PCR was performed on a QuantStudio3 PCR system (ThermoFisher Scientific, Waltham, MA, USA). The miRCURY LNA (50) miRNA Custom PCR Panel (QIAGEN, Germantown, MD, USA) was used to quantify the pre-selected miRNAs (**Table 1**). Each sample was measured on a separate plate; in each plate, 44 miRNAs, RNUB6, UniSp6 (cDNA synthesis control), UniSp3 (RT-qPCR positive control and inter-plate calibrator (IPC), and non-template control (negative control) were measured. The cDNA dilution was 1:80, according to the manufacturer’s instructions. Relative miRNA expression levels

**Table 1:** The selected miRNAs for miRNA Custom PCR Panel.

	miRNA ID	MiRCury Assay cat. No.	Sequence
1	miR-192-5p	YP00204099	CUGACCUAUGAAUUGACAGCC
2	miR-20b	YP00205943	CAAAGUGCUCACAGUGCAGGUA
3	miR-18a-5p	YP02100185	UAAGGUGCAUCUAGUGCAGAUA
4	miR-20a-5p	YP00204292	UAAAGUGCUUAUAGUGCAGGUAG
5	miR-17-3p	YP00206008	ACUGCAGUGAAGGCACUUGUAG
6	miR-106a	YP02107906	AAAGUGCUUACAGUGCAGGUAG
7	miR-19b	YP02105441	UGUGCAAAUCCAUGCAAAACUG
8	miR-425-5p	YP00204337	AAUGACACGAUCACUCCCGUUGA
9	miR-664	YP02114877	UGGGCUAGGAAAAAUGAUUGGA
10	miR-92b-3p	YP00204384	UAUUGCACUCGUCCCGGCCUCC
11	miR-92a-3p	YP00204258	UAUUGCACUUGUCCCGGCCUGU
12	miR-144	YP02107159	UACAGUAUAGAUGAUGUACUAG
13	miR-1842	YP02105706	UGGCUCUGCGAGGUCAGCUCA
14	miR-1840	YP02102662	UCACGUGACGGGCCUCGGCG
15	miR-451a	YP02119305	AAACCGUUACCAUUACUGAGUU
16	miR-31	YP02119121	AGGCAAGAUGCUGGCAUAGCUGU
17	miR-34a-5p	YP00204486	UGGCAGUGUCUUAGCUGGUUGU
18	miR-363	YP02110319	AAUUGCACGGUAUCCAUCUGUAA
19	miR-1839-5p	YP00205327	AAGGUAGAUAGAACAGGUCUUG
20	miR-217	YP00204010	UACUGCAUCAGGAACUGAUUGGAU
21	miR-574-3p	YP00206011	CACGCUCAUGCACACACCCACA
22	miR-139	YP02100609	UGGAGACGCGGCCCUGUUGGAA
23	miR-152-3p	YP00204294	UCAGUGCAUGACAGAACUUGG
24	miR-146a-5p	YP00204688	UGAGAACUGAAUUCCAUGGGUU
25	miR-151a-5p	YP00204007	UCGAGGAGCUCACAGUCUAGU
26	miR-216b-5p	YP00204289	AAAUCUCUGCAGGCAAAUGUGA
27	miR-150-5p	YP00204660	UCUCCCAACCCUUGUACCAGUG
28	miR-379-5p	YP00205658	UGGUAGACUAUGGAACGUAGG
29	miR-885-5p	YP00204473	UCCAUUACACUACCCUGCCUCU
30	miR-504-5p	YP00204396	AGACCCUGGUCUGCACUCUAUC
31	miR-132	YP02104207	UAACAGUCUACAGCCAUGGUCGC
32	miR-29c-3p	YP00204729	UAGCACCAUUUGAAAUCGGUUA
33	miR-128-3p	YP00205995	UCACAGUGAACCGGUCUCUUU
34	miR-378a-3p	YP00205946	ACUGGACUUGGAGUCAGAAGGC
35	miR-98-5p	YP00204640	UGAGGUAGUAAGUUGUAUUGUU
36	miR-218-5p	YP00206034	UUGUGCUUGAUCUAACCAUGU
37	miR-21-5p	YP00204230	UAGCUUAUCAGACUGAUGUUGA
38	miR-129-5p	YP00204534	CUUUUUGCGGUCUGGGCUUGC
39	miR-101	YP00205955	UACAGUACUGUGAUAACUGA
40	miR-15a	YP02103582	UAGCAGCACAUAAUGGUUUGU
41	miR-22-3p	YP00204606	AAGCUGCCAGUUGAAGAACUGU
42	miR-450a	YP02116559	UUUUUGCGAUGUGUUCCUAAUA
43	miR-99a-5p	YP00205945	AACCCGUAGAUCCGAUCUUGU
44	miR-361-5p	YP00206054	UUAUCAGAAUCUCCAGGGGUAC
45	UniSp6	YP00203954	
46	UniSP3	YP02119288	
47	U6 snRNA	YP00203907	

RT-qPCR normalization for reducing technical variation was performed to validate the were calculated by the Livak method 2^-ΔΔcq^ (51).

#### 2.5.1 Reference miRNAs selection

The applied criteria in selecting reference candidates for normalizing the exported calibrated quantification cycles (Cq) are shown (**Supplementary Table 7**). First, a set of miRNA candidates was selected manually based on the lowest coefficient of variation (CV%) (52, 53), followed by using RefFinder (https://www.heartcure.com.au/reffinder/) (54). This web-based tool uses an ensemble approach based on several computational algorithms (geNorm (55), NormFinder (56), the comparative Delta-Cq method (57), and BestKeeper (58)) to rank potential reference miRNAs according to their stability of expression as demonstrated. Each program ranks and ascribes weights to the tested miRNAs. The geometric mean of those weights is calculated and used as the final weight (**Table 2**).

**Table 2:** Overview of selecting potential reference miRNAs from healthy and DLBCL sample processes.

Genes	CV%	RefFinder incorporated algorithms				
		Δ Cq	Normfinder	geNorm	BestKeeper	RefFinder
		Ave.SD	SV	SV	SD	GM
miR-361-5p	1.819	1.07	0.396	0.507	0.43	1.32
miR-101	1.845	1.11	0.503	0.507	0.38	1.86
miR-29c-3p	1.803	1.12	0.491	0.63	0.28	2.06
miR-22-3p	3.259	1.19	0.602	0.904	0.7	4.47
miR-378a-3p	3.319	1.42	1.098	0.774	0.7	4.47
miR-129-5p	3.638	1.53	1.224	1.036	1.2	6.24
miR99a-5p	5.56	1.66	1.402	1.203	1.03	6.74
U6 snRNA	6.96	1.88	1.661	1.371	1.23	8

A set of miRNA candidates was selected based on the coefficient of variation (CV%) followed by RefFinder (geNorm, NormFinder, and the comparative Delta-Cq method). Using different statistical approaches, they ranked from higher to lower stability (top to bottom). The ranking of candidate reference miRNAs results showed that miR-361-5p, miR-101and miR-29c-3p are the top three stably expressed miRNAs that can be used as reference miRNAs for normalization of the experimental data. Ave.SD, average standard deviation; GM, geometric mean; SD, standard deviation; SV, stability value.

#### 2.5.2 Statistical *a*nalysis of RT-PCR

All statistical analyses were performed using R version 4.1.2. Unpaired parametric Welch’s 2- sample t-test was used to explore the differences in the fold-change (2^-ΔΔcq^) in the expression of each miRNA in the DLCBL and healthy groups. To address the multiple testing problem, which results in increased false-positive rates, P-values were corrected (59) using Benjamini–Hochberg (FDR) with P-values treated according to their ranks (60). The adjusted P-values less than 0.05 were used to indicate significance.

## 3 Results

### 3.1 RNA extraction and small RNA sequencing analysis

The mean concertation of the total RNA extracted was 1354.33 ± 759.65 ng/µL measured by UV spectrophotometer. The ratio 260/280nm absorbance ratio was 2.1 ± 0.01 (**Supplementary Table 2**).

**Figure 1:**
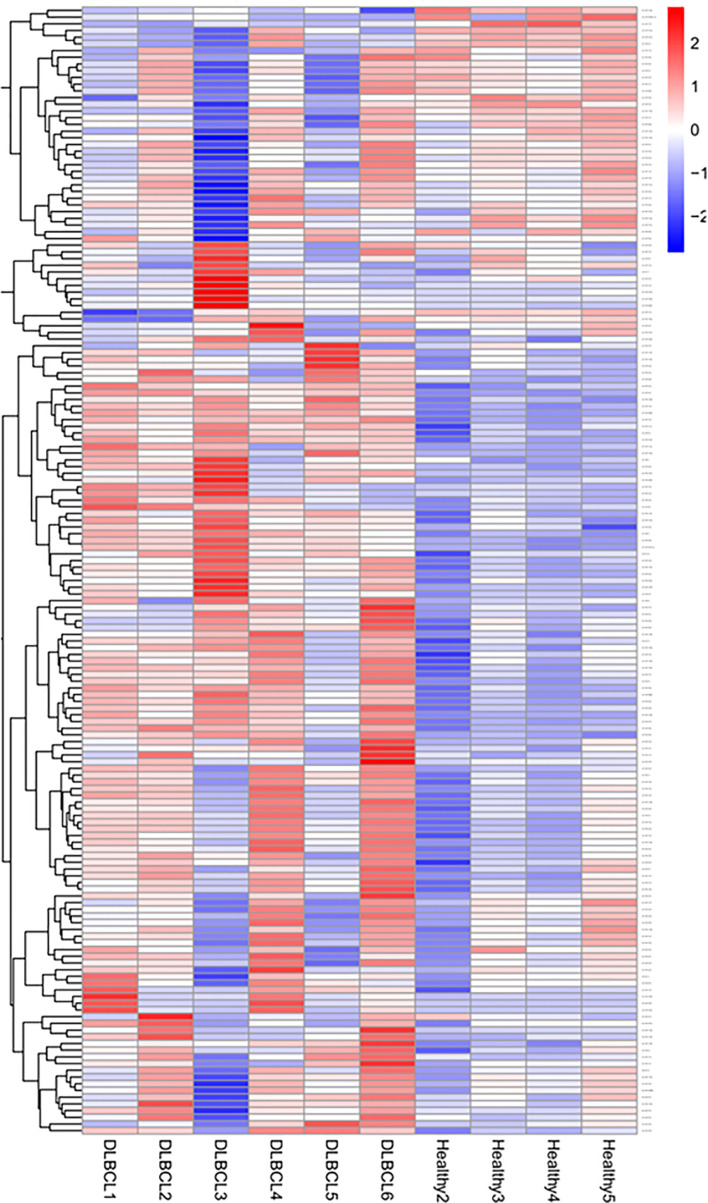
sRNA-Seq heatmap of the expressed miRNAs (DEMs). Red represents high expression; blue represents low expression. The color intensity from red to blue represents the log10 (TPM+1) value from large to small.

**Figure 2:**
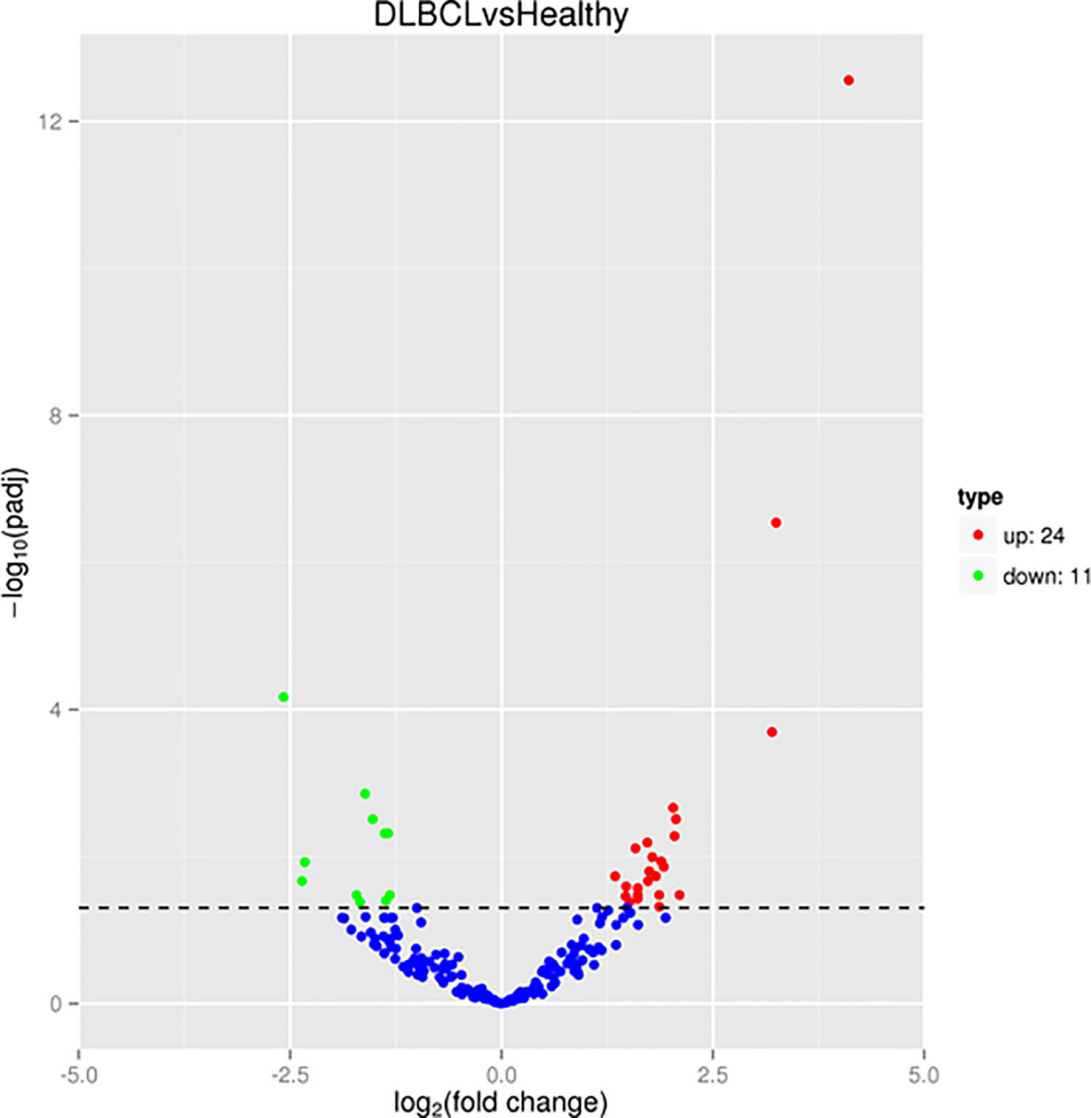
sRNA-Seq volcano plot of the differentially expressed miRNAs (DEMs). Red represents high expression; green represents low expression.

The mean number of sRNA reads was 475,87,913, with an average of 96.75% of sRNA retained after quality trimming and adapter removal for all samples. A summary of the overall cleaning process and mapping statistics of the reads are shown (**Supplementary Table 5**). A total of 320 miRNAs was detected and the cluster analysis of all the differentially expressed miRNAs from the healthy and DLBCL samples are shown on the criteria of a fold change ≥2 or ≤- 2 and *p-value <*0.05 and presented in a heat map (**Figure 1**). Twenty-four miRNAs were upregulated, and 11 downregulated in DLBCL relative to normal lymph nodes (35 total DEMs) are presented in a volcano blot (**Figure 2**).

### 3.2 Gene ontology analysis of the differentially expressed miRNAs

**Figure 3:**
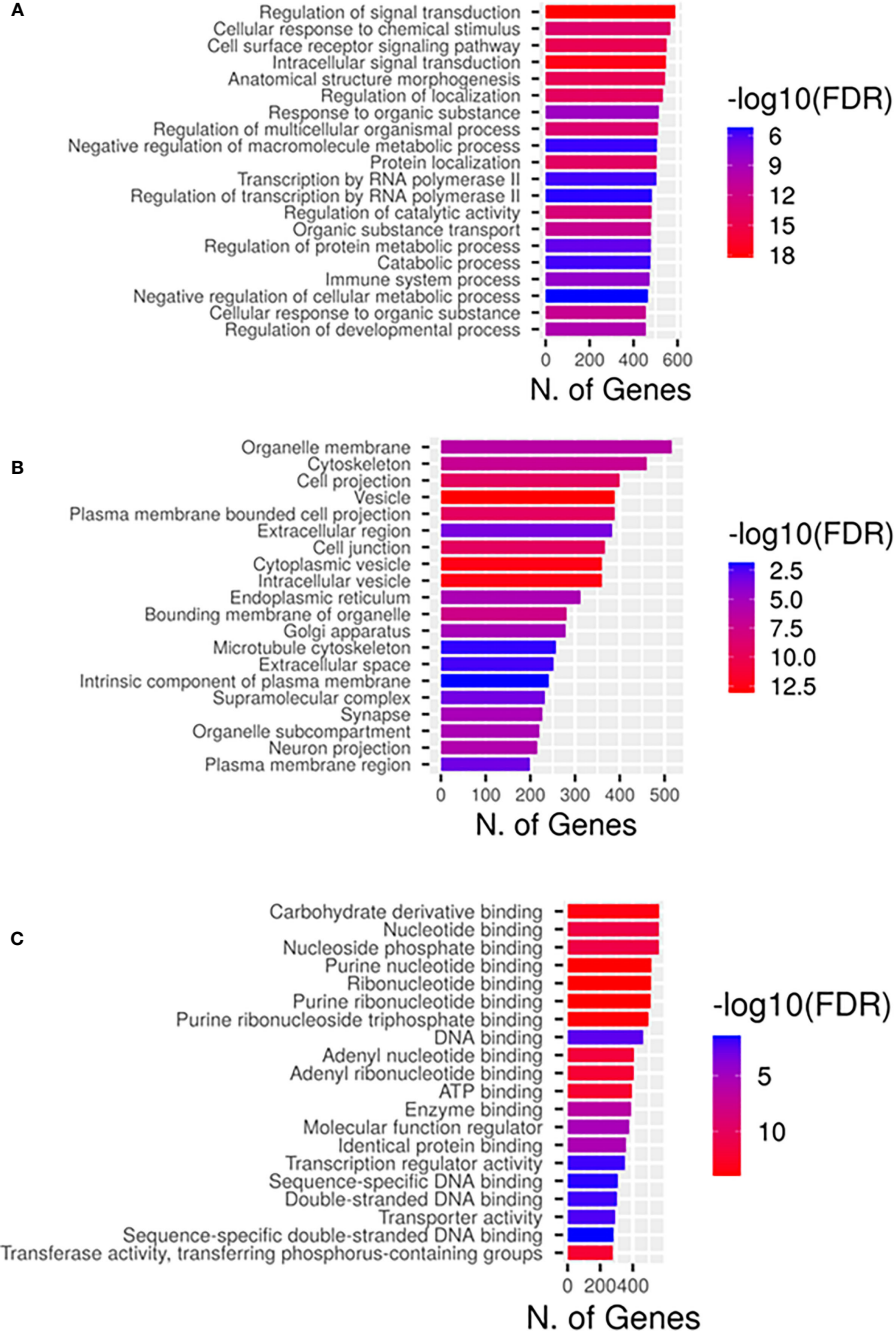
Gene ontological classifications of the differentially expressed miRNAs (DEMs) targets by sRNA-Seq between DLBCL and healthy group. The differentially expressed genes are grouped into three GO terms, **(A)** Biological process, **(B)** Cellular components, and **(C)** Molecular functions. The abscissa represents the number of annotated genes enriched primarily in each GO term.

**Figure 4:**
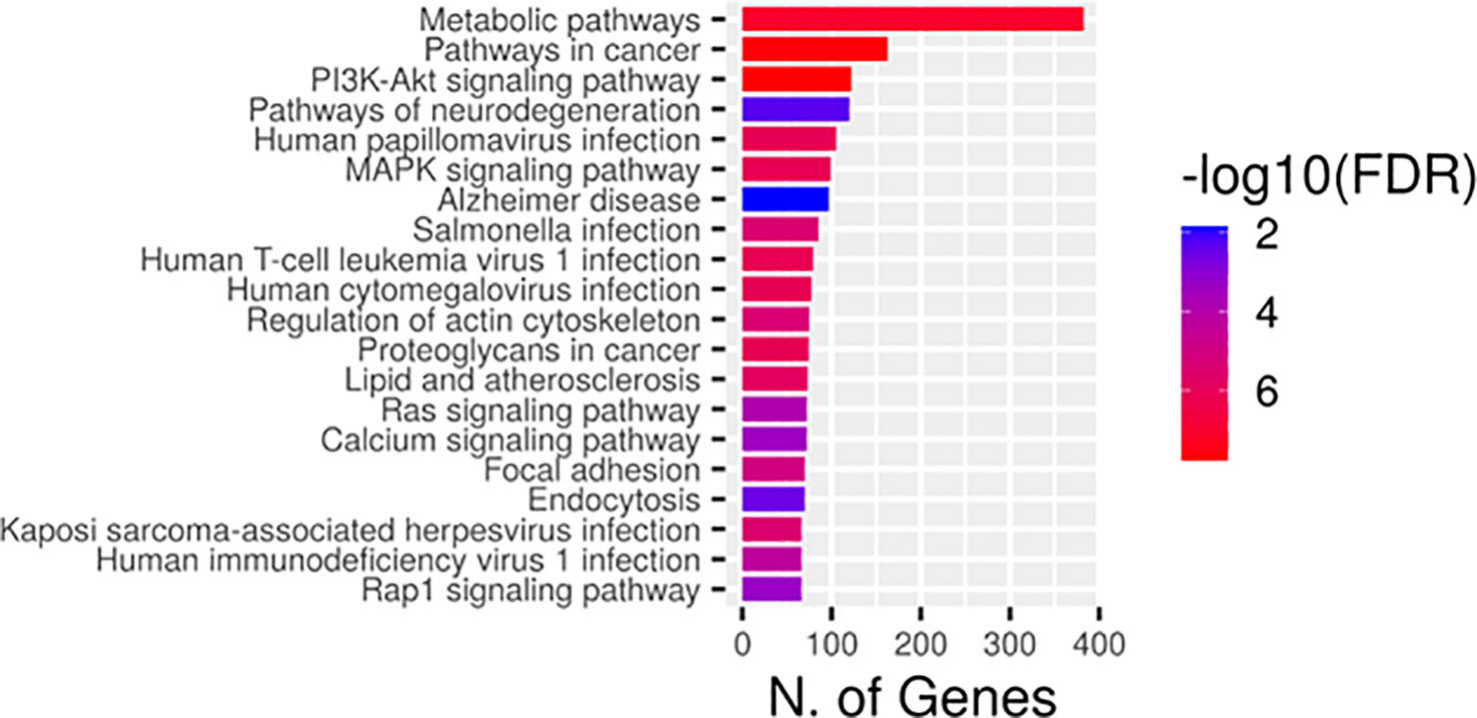
Kyoto Encyclopedia of Genes and Genomes (KEGG) annotation of the differentially expressed miRNAs (DEMs) in DLBCL. Each circle represented a KEGG pathway, the name of which was shown on the left legend. Abscissa showed the number of genes enriched in each pathway. The bigger the fold enrichment is, the more significant the pathway. The color of the barplot represented negative log_10_ of the false discover rate (FDR) (red: high, blue: low).

Gene ontology enrichment showed that the predicted top 20 GO terms target genes regulated by the DEMs are mainly implicated in the regulation of signal transduction biological process (**Figure 3A**), cytoskeleton and cytoplasmic vesicle cellular compartments (**Figure 3B**), and carbohydrate and nucleotide binding molecular function (**Figure 3C**). Pathway enrichment KEGG (**Figure 4**) uncovered that the DEMs predicted targets were primarily involved in metabolic pathways; Pathways in cancer (**Figure S1**), PI3K-Akt Pathway (**Figure S2**), and MAPK signaling pathway (**Figure S3**). Reactome pathways revealed DEMs targets mainly involved in the immune system, small molecule transport, and cytokine signaling in the immune system (**Figure S4**). In contrast, for PANTHER and wikiPathways, the integrin signaling pathway (**Figures S5A, B**) and MAPK signaling pathway (**Figure S3**) had the most prominent DEMs predicted targets, respectively.

### 3.3 Quantitative PCR analysis

The mean concertation of the total RNA extracted was 193.25 ± 124.2 ng/µL as measured by UV spectrophotometry. The 260/280 nm absorbance ratio was 1.966 ± 0.0302, while the mean concentration of miRNAs measured by spectrofluorometer was 67.622 ± 46.8 ng/µL, as shown in (**Supplementary Table 2**).

**Figure 5:**
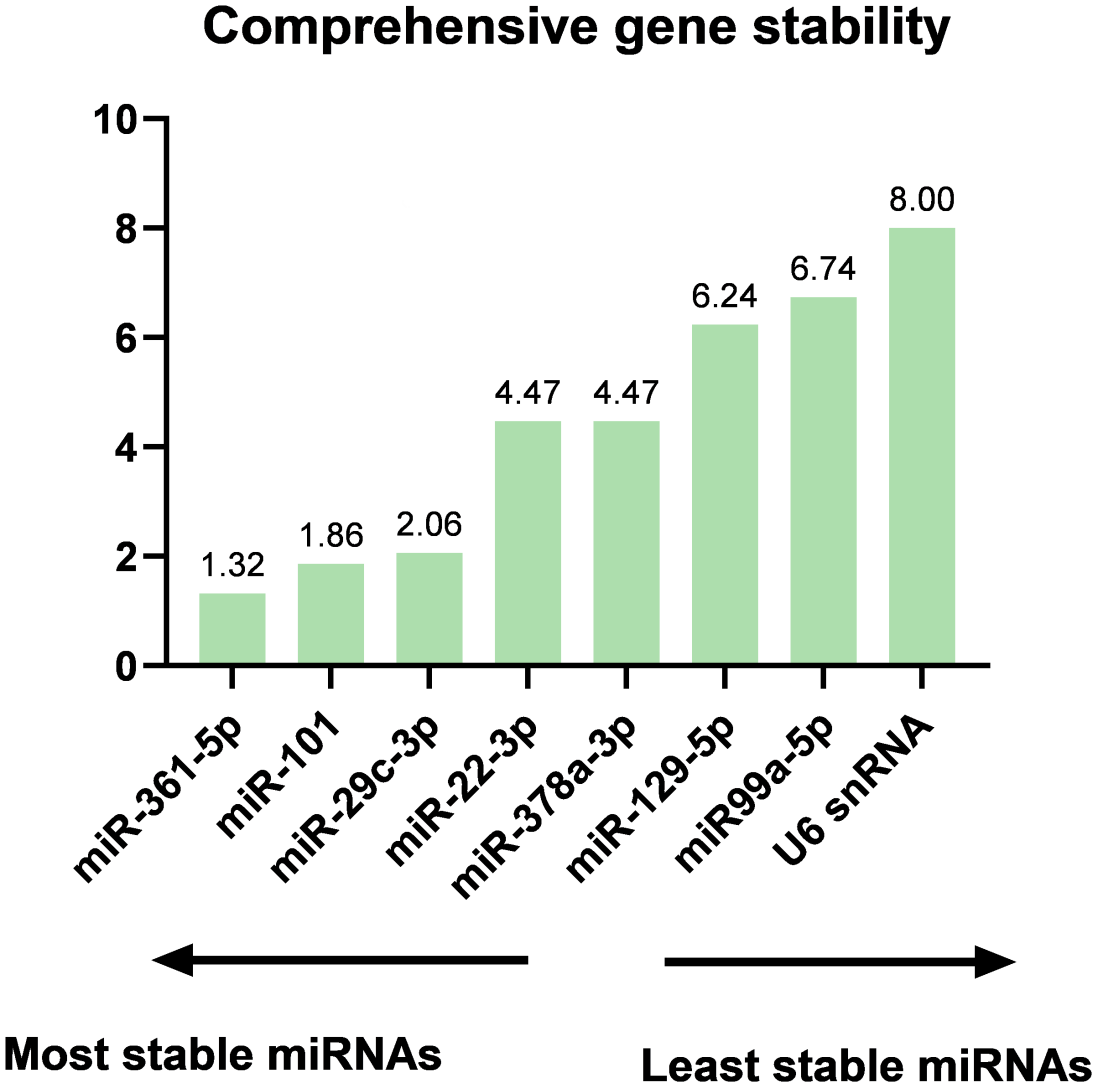
The ranking of candidate reference genes is based on the geometric mean of the ranking values by RefFinder. The results showed that miR-361-5p, miR-101, and miR-29c-3p are the top three stably expressed miRNAs that can be used as reference miRNAs for the normalization of the experimental data.

**Figure 6:**
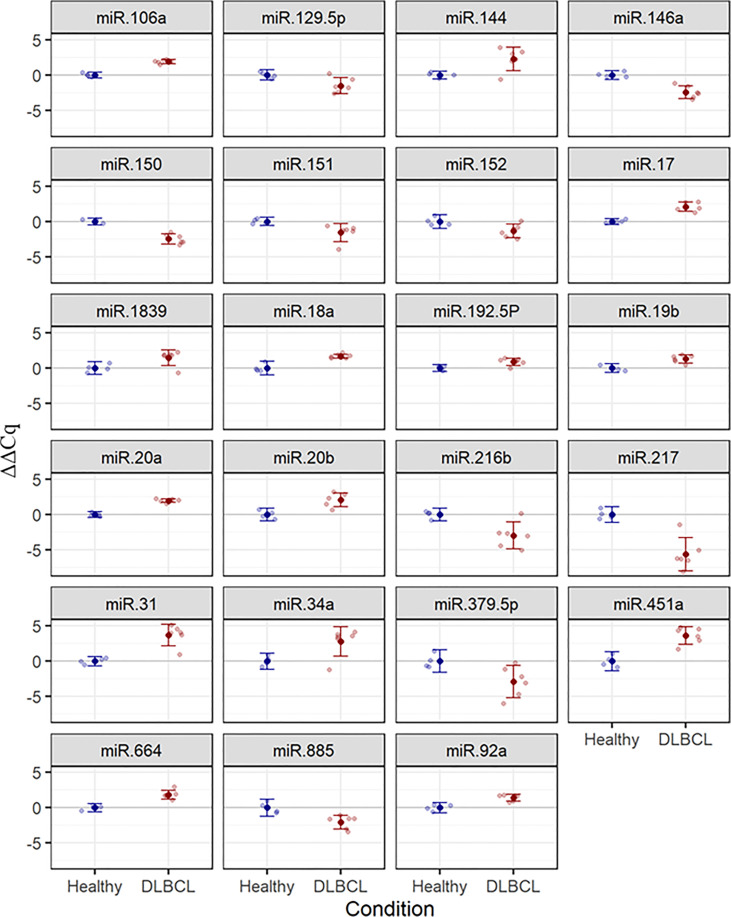
Estimated mean (± 95%) ΔΔCq values for expression of 23 selected miRNAs in healthy (n = 4) and DLBCL (n = 6) groups. Lighter points show individual samples. FDR-adjusted *P-values* for two-sample t-tests are ≤ 0.048 in all cases.

**Figure 7:**
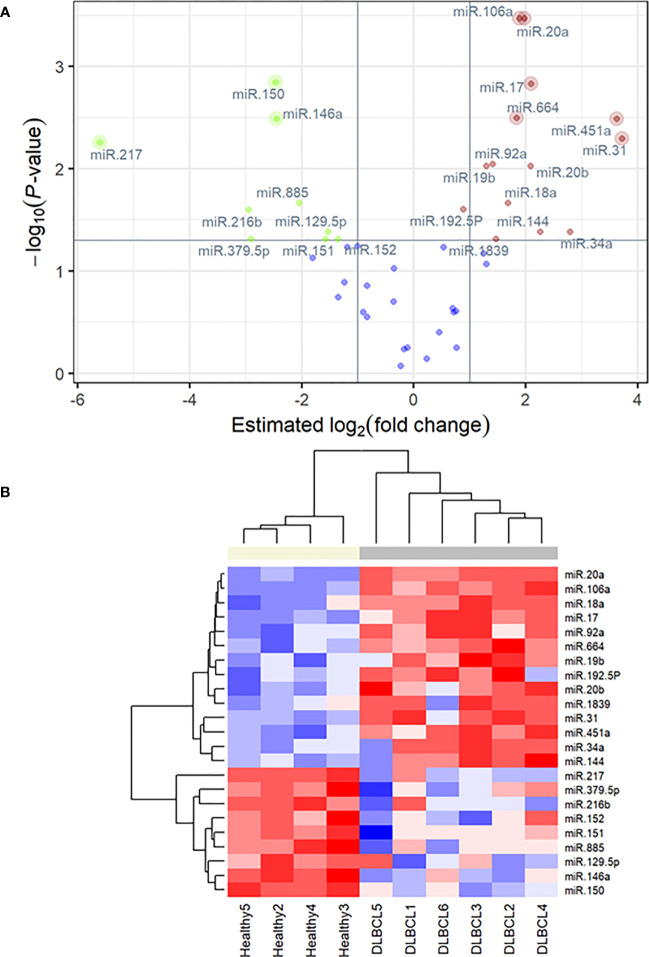
**(A)** q-PCR volcano plot showing FDR-adjusted, negative log10 *P-value* versus the estimated difference between mean ΔΔCq values in the DLBCL group (n = 6) relative to the healthy group (n = 4) for 45 micro-RNA markers. Labeled points highlight statistically significant differences in expression. Points with lighter outer rings are also significant using a more conservative Bonferroni-Holm correction. **(B)** qPCR heatmap of 23 differentially expressed miRNAs identified as significant in FDR-corrected two-sample t-tests. The colored bar at the top of the plot distinguishes normal subjects (beige) from cancer subjects (grey).

The cDNA synthesis control, UniSp6, was amplified at 20.9 Cq ( ± 0.45) in all samples. The qPCR positive inter-plate control, UniSp3, was amplified in all samples, and Cqs calibrated accordingly for all samples to 21.638 to limit run-to-run deviation. The average Cq values for each tested miRNA and RefFinder recommended three references (miR-361-5p, miR-101, and miR-29c-3p) with their geometric mean ranking values (1.32, 1.86, and 2.06) and average Cq of 28.12, 26.61, and 23.86, respectively are shown (**Figure 5**, **Supplementary Table 7**). The relative expression in DLBCL and healthy group of each miRNA was analyzed individually compared to the geometric mean of the expression level of miR-361-5p, miR-101, and miR-29c-3p in each sample by Welch’s 2-sample t-test (**Figure 6** and **Table 3**). We confirmed the miRNA sequence result for 23/35 DEMs after applying cut-off FDR <0.05 and fold change < 2 (**Figures 7A, B**). Fourteen of the 23 DEMs were upregulated, and nine were downregulated in DLBCL relative to the normal lymph node. Specifically, we observed the dysregulation of 23 miRNAs that regulate two axes in DLBCL progression. The first axis promotes uncontrolled lymphoid proliferation, cellular growth, and apoptosis inhibition; this axis is regulated by oncomiRs (18, 19, 61), such as miR-17/92, miR-31, miR-34a, miR-106a, miR-451,miR-192-5p, and miR-1839, which we found to be overexpressed. The second axis is associated with the downregulation of the tumor suppressor miRNAs (62–64), such as miR-146a, miR-150, miR-151, miR-152, miR-216b, miR-217, and miR-885.

**Table 3:** Two-sample t-test results for differences in ΔΔCq between Healthy (n = 4) and DLBCL (n = 6) groups in 44 miRNAs, with FDR adjustment (lowest *P-values* first).

miRNA	difference	Mean	Mean	statistic	P-value	FDR
		(DLBCL)	(Healthy)			
miR-20a	1.973	1.973	1.15E-10	12.039	0	0
miR-106a	1.89	1.89	3.93E-11	10.945	0	0
miR-150	-2.468	-2.468	-1.70E-10	-7.714	0	0.001
miR-17	2.089	2.089	1.07E-10	7.5	0	0.001
miR-664	1.837	1.837	2.02E-11	5.94	0	0.003
miR-146a	-2.449	-2.449	-1.70E-10	-6.012	0	0.003
miR-451a	3.625	3.625	4.65E-11	5.672	0.001	0.003
mirR-31	3.716	3.716	-5.34E-12	5.959	0.001	0.005
miR-217	-5.607	-5.607	1.81E-10	-5.674	0.001	0.006
miR-92a	1.404	1.404	2.39E-11	4.817	0.002	0.009
miR-20b	2.08	2.08	1.40E-11	4.386	0.002	0.009
miR-19b	1.291	1.291	4.11E-12	4.358	0.002	0.009
miR-885	-2.045	-2.045	1.52E-10	-3.747	0.006	0.022
miR-18a	1.681	1.681	3.65E-11	5.275	0.007	0.022
miR-192-5P	0.879	0.879	1.35E-11	3.487	0.008	0.025
miR-216b	-2.947	-2.947	2.32E-11	-3.725	0.009	0.025
miR-144	2.258	2.258	7.58E-11	3.359	0.016	0.041
miR-129-5p	-1.533	-1.533	1.85E-10	-3.08	0.017	0.041
miR-34a	2.793	2.793	-4.98E-11	3.133	0.017	0.041
miR379-5p	-2.911	-2.911	2.17E-10	-2.847	0.023	0.048
miR-152	-1.35	-1.35	2.48E-10	-2.774	0.024	0.048
miR-151	-1.573	-1.573	2.42E-10	-2.995	0.023	0.048
miR-1839	1.463	1.463	-2.22E-10	2.777	0.025	0.048
miR-21-5p	-1.013	-1.013	4.06E-11	-2.649	0.03	0.057
miR-29c-3p	0.526	0.526	-2.80E-10	2.576	0.033	0.059
miR-132	-1.186	-1.186	2.77E-10	-2.571	0.034	0.059
miR-1842	1.243	1.243	4.05E-10	2.818	0.04	0.067
miR-574-3p	-1.805	-1.805	2.84E-12	-2.43	0.046	0.074
miR-363	1.295	1.295	-1.35E-10	2.349	0.055	0.086
miR-361	-0.353	-0.353	9.62E-12	-2.322	0.062	0.093
miR-504-5p	-1.243	-1.243	2.48E-10	-2.042	0.088	0.128
miR-22-3p	-0.835	-0.835	-2.65E-10	-1.939	0.099	0.139
miR99a-5p	-1.353	-1.353	4.02E-10	-1.76	0.131	0.179
miR-98-5P	-0.368	-0.368	1.74E-10	-1.754	0.149	0.198
miR-378a-3p	0.69	0.69	-1.26E-10	1.479	0.179	0.23
miR-425-5p	0.749	0.749	-1.25E-10	1.443	0.198	0.247
miR-450a	-0.907	-0.907	5.06E-11	-1.41	0.207	0.252
miR-1840	0.707	0.707	2.11E-11	1.438	0.213	0.252
miR-218-5p	-0.834	-0.834	2.46E-10	-1.263	0.242	0.28
miR-128-3p	0.453	0.453	4.58E-11	0.992	0.353	0.397
miR-15a	-0.119	-0.119	1.91E-10	-0.674	0.52	0.558
U6 snRNA	0.765	0.765	9.85E-11	0.675	0.521	0.558
miR-101	-0.173	-0.173	1.96E-10	-0.639	0.551	0.577
miR-92b-3p	0.23	0.23	1.09E-10	0.403	0.699	0.715
miR-139	-0.238	-0.238	-1.82E-10	-0.199	0.848	0.848

## 4 Discussion

Small RNA-Seq has made distinctive contributions to discovering and quantifying miRNAs, investigating their differential expression contributing to tumorigenesis, and identifying highly similar miRNA family members that vary by a single nucleotide. The main focus of this research is to validate the sRNA-Seq results using RT-qPCR and explore the roles of miRNAs and different signaling pathways.

The DEMs observed herein are mainly predicted to regulate the RAS signaling pathways. The activation of receptor tyrosine kinase stimulates RAS to initiate two major downstream pathways: the phosphatidylinositol-3-kinase (PI3K)/serine-threonine kinases (AKT) (65) and mitogen-activated protein kinases (MAPK). Those, in turn, may result in the activation of C-Myc and transcription factor NF-κB, also enriched by the DEMs. Moreover, two miRNAs involved in the p53 pathway are reported. The disturbed regulation of these pathways may stimulate lymphoid cell proliferation growth and inhibition of apoptosis, as shown in other cancers (65–67).

The PI3K/AKT signaling cascade is one of the most significant intracellular signaling pathways, commonly activated in various malignancies (68). In health, the PI3K/AKT signaling pathway involves multiple biological functions, including cell growth and fate. Disruption of this pathway affects cell growth, development, metabolic activity, and cytoskeletal remodeling, resulting in cancer cell survival and therapy resistance (69–71). We found that miR-151, miR-192-5p, miR-152, and miR-885-5p are involved in this pathway and are dysregulated in canine DLBCL compared to lymph node tissue from healthy dogs. The miR-151 downregulation is also reported in other cancers, including Epstein-Barr virus-induced DLBCL (72). We also verified the downregulation of another tumor suppressor, miR-152, which exerts its function through its antiproliferative activity (73), repressing the PI3K signaling pathway (74). The phosphatase and tensin homolog (PTEN) inhibit the PI3K/AKT pathway with vital anticancer activity; we report the downregulation of miR-885-p, reported to activate the PI3K/AKT pathway by inhibiting PTEN (75). We also report the upregulation of miR-192-5p, which is also significantly overexpressed in the low-grade B-cell lymphoma, Waldenstrom Macroglobulinemia (76, 77), suggesting an oncogenic function. In addition, miR-144 was upregulated in canine DLBCL. The role is miR144-3p was investigated in other cancers, where the upregulation of miR-144-3p inhibits PTEN, promoting apoptosis of cancer cells (78–80). Noteworthy miR-451 is also upregulated in this study in canine DLBCL. The tuning function of both miRNAs has been reported in tumorigenesis (81), and its expression is reportedly altered in various cancers (82–84).

The other downstream arm of the RAS pathway is the activation of MAPK. These protein kinases auto-phosphorylate their serine and threonine residues to induce or inhibit their target (85), regulating essential biological functions such as cellular division, death, oxidative stress, and immune response (86, 87). We found that miR-146a and miR-31, involved in this pathway, are dysregulated in canine DLBCL compared to lymph node tissue from healthy dogs. The expression of miR-146a is altered in various cancers (88, 89) and reportedly targets IRAK1 and TRAF6, leading to the inactivation of the inflammatory, tumorigenic NF-kB signaling pathway (90, 91), apoptosis stimulation, and cancer cell proliferation reduction (92). Function studies are needed to determine the role of miR-146a in canine DLBCL. We also verified miR-31 overexpression in the DLBCL group compared to controls. The intricate dual function of miR-31 has been studied in various cancers (93–97). As a tumor suppressor, miR-31 affects genes implicated in PI3K/AKT and DNA repair, while its upregulation activates other pathways, including WNT, Hippo, and NF-κB (98). Given its upregulation in this study, miR-31 may act as an oncomiR in canine DLBC lymphoma; however, further studies are needed to elucidate its regulatory role in the carcinogenesis of this tumor.

Activation of signal transduction pathways such as RAS-PI3K/AKT and RAS-MAPK will converge on the nucleus to activate transcription factors responsible for diverse cellular functions. The MYC proto-oncogene rapidly responds to the stimulation of the RAS-MAPK pathway. The C-Myc signaling pathway regulates critical cellular functions, including division, diversification, cell signaling, metabolic activity, and cell death. Substantial evidence supports that abnormal MYC expression drives tumor onset and progression (99) and links it to all defining features of cancer (100, 101). Notably, we verified the overexpression of the oncogenic polycistronic cluster miR-17/92 (miR-17, miR-18a, miR-19b, miR-20a, and miR-92a), also known as oncomiR-1 (102, 103) and its paralogue miR-106a/363 (miR-106a, and miR-92a) (104) in canine DLBCL compared to healthy lymph nodes. OncomiR-1 is frequently dysregulated in canine and human DLBCL (105–108). OncomiR-1 is a direct downstream target of Myc (109). Myc regulates and activates oncomiR-1 that, in turn, targets and inhibits PTEN (110–112), consequently activating AKT (113, 114), as well as attenuates the proapoptotic protein BimL (115, 116) or transcription factor E2F (117, 118) hence, preventing apoptosis and DNA repair. Strong evidence suggests that the aberrant expression of oncomiR-1 leads to cancer development, including B-cell lymphoma in humans (119–121) and murine models (102, 103, 122). In line with previous studies, we observed the overexpression of miR-19b/20a (111, 123), which is known for repressing apoptosis and inducing malignant transformation by activating the mammalian target of the rapamycin (mTOR) pathway (124–126) and by suppressing PTEN (123, 127, 128).

Along with our previous study (129), sRNA-Seq and RT-qPCR quantification verified the upregulation of miR-34a in canine DLBCL (120, 129–131). This finding does not corroborate studies in human cancers where miR-34 is downregulated (132). This finding is explained by Christofferson and collaborators (2010), who elucidated the role of miR-34a in cellular senescence where it was activated independently of p53, the upstream regulator, to inhibit MYC through utilizing another pathway that involves the ETS transcription factor ELK1 (133, 134). We also report the downregulation of the tumor suppressor miR-150, a regulator of MYC (135). The effect of increased expression of miR-150 in reducing tumor growth has been investigated in many cancers (136–138). Xiao and collaborators (2016) investigated the regulatory role between miR-150 and c-Myb in B-cell development *in vivo* (139, 140) and concluded that miR-150 is expressed in mature lymphocytes and directly targets and activates c-Myc (141). The interplay between miR-150 and c-Myb can result in a wide range of changes, such as a severe block of B-cell development, deletion of the c-Myb, or one of the mature B-cell subsets expansion in case of miR-150 deficiency (142). Function studies of miR-150 are warranted in canine DLBCL.

Nuclear factor-κB (NF-κB) belongs to a family of inducible transcription factors that regulate many genes involved in immune and inflammatory responses. We report the downregulation of miR-217 in canine DLBCL, in agreement with what is reported in many cancers (143–145). Although the function of miR-217 is not fully established in B-cell lymphomas, the overexpression of miR-217-5p significantly decreased breast cancer cell proliferation, invasion, migration, and suppressed epithelial-to-mesenchymal transition due to the inhibition of the NF-κB signaling pathway by directly targeting metadherin (MTDH) (146) which is also known as astrocyte elevated gene 1 (AEG1) or LYRIC (Lysine Rich CEACAM1) (147). In laryngeal cancer, miR-217 exerts an anti-metastatic and antiproliferative effect via repression of its downstream target genes MTDH and the programmed cell death protein 1-ligand 1 (PD1-L1) (148) at the translational level. Interestingly, the increased expression of MTDH may also be driven by the PI3K/ARK, MAPK, Myc, and Wnt/β-catenin pathways (147, 149). It is noteworthy that given the multiple pathways interactions, oncogenic roles, and involvement in the chemoresistance of MTDH/LYRIC, there is an increased interest in investigating this molecule as a potential therapeutic target in cancer (147).

We also report two dysregulated miRNAs in canine DLBCL involved in the p53 pathway: miR-664 (upregulated) and miR-379-5p (downregulated). The p53 protein responds to a wide range of cellular stress signals (150) by regulating cell cycle and DNA repair, enhancing cell senescence (151) and apoptosis, and altering the cellular environment by changing the extracellular matrix and angiogenesis in a specific tissue location (152). Thus, the p53 pathway is tightly regulated by positive or negative feedback loops (152). The upregulation of miR-664 is also reported in human lung cancer cells (153), osteosarcoma (154), and hepatocellular carcinoma, where it was associated with poor prognosis (155). In squamous cell carcinoma, miR-664 upregulation promotes cell proliferation, migration, and invasion by targeting interferon regulatory factor 2 (IRF2), which inhibits p53 expression (156). Conversely, a tumor suppressor function has been shown in cutaneous malignant melanoma (157) and breast cancer (158). The oncogenic function is canine DLBCL is likely, but studies are needed to determine if similar mechanisms and targets apply to this tumor. The predicted dysregulated miRNA-mediated pathways in canine DLBCL need confirmation of the inverse correlation to important targets in tumor tissues to substantiate the pathway analysis results. Thus, our subsequent studies include the integration of transcriptome and proteome data.

Our study shows the downregulation of miR-129-5p in the DLBCL samples. Consistent results have been observed in other relevant research studies; gastric cancer (159, 160), colorectal cancer (161, 162), liver cancer (163), endometrial cancer (164), and esophageal cancer (165, 166). The miR-129 promoter hypermethylation (162, 167) allows the overexpression of SOX4 and, subsequently, tumor initiation, progression, and metastasis (164, 167)

Limitations of the study include a variation in the miRNA expression observed within the DLBCL group (**Figure 1**, **Supplementary Table 1**); this is explained by the heterogeneous nature of lymphomas even within the same type and subtypes, and corroborated by differences in mitotic index, staging, and progression-free survival time (168). Two biologically distinct groups are observed when focusing on validated DEMs between healthy and DLBCL patients (**Figure 7B**). Despite the heterogeneity of this disease, this set of 23 miRNAs can differentiate DLBCL from healthy patients. Further analyses with a larger cohort of DLBCL cases are needed to determine if DEMs within DLBCL patients may be markers to subtype or infer prognostication.

Unlike human studies, our study compared fresh frozen lymph nodes from canine DLBCL patients to healthy controls instead of reactive lymph nodes. Biopsy for histopathology of reactive lymph nodes is not commonly performed in a clinical setting in dogs due to concerns about the dog’s comfort, the invasiveness of the procedure, the possibility of complications, and the dog owners’ decision. A few studies on human patients revealed altered expression of miRNAs in reactive lymph nodes (169–173). These miRNAs may regulate immune cell activities and lead to lymph node reactivity. Thus, our subsequent studies will focus on a large prospective cohort of patients exploring different sample types (i.e., fine-needle aspirate) and the ability of the reported DEMs to discriminate DLBCL from other lymphomas and reactive lymph nodes.

## 5 Conclusions

Identifying biomarkers that allow earlier diagnosis of DLBCL is critical to improving treatment outcomes. Our study revealed 24 upregulated and 11 downregulated miRNAs in canine DLBCL relative to normal lymph nodes by sRNA-Seq, totaling 35 DEMs. From those, 23 DEMS were validated by RT-qPCR, 14 were upregulated, and nine were downregulated. Our results hold the promise of miRNAs as diagnostic biomarkers for DLBCL. Many DEMs identified herein are reportedly dysregulated in human DLBCL, such as the well-described miR-17/92 cluster, and miRNAs miR-106a, miR-451a, and miR-31 that were upregulated. Moreover, miR-150, miR-151, and miR-152 are downregulated in DLBCL in dogs and humans. Two DEMs (miR-216b and miR-1839) are reported for the first time to be differentially expressed between DLBCL and healthy controls in dogs. KEGG enrichment pathway analysis showed that the predicted target genes regulated by DEMs are mainly implicated in metabolic pathways, pathways in cancer, PI3K-AKT pathway, and MAPK signaling pathway. Our study serves as a building block for subsequent studies that will validate the diagnostic utility of this set of DEMs in a larger, prospective cohort of patients. The ability of this set of DEMs to distinguish DLBCL from other lymphoma subtypes and reactive lymph nodes will also be explored in future work. Our study serves as a guideline for subsequent experimental studies to determine the targets and functions of these altered miRNAs in canine DLBCL.

## Data availability statement

The original contributions presented in the study are publicly available. This data can be found here: https://www.ncbi.nlm.nih.gov/geo/query/acc.cgi?acc=GSE240893.

## Ethics statement

The animal studies were approved by The Purdue Animal Care and Use Committee (protocols #1708001607 and #1111000308). The studies were conducted in accordance with the local legislation and institutional requirements. Written informed consent was obtained from the owners for the participation of their animals in this study.

## Author contributions

Conceptualization: NE, MC, and AS. Data curation: NE and MG. Formal analysis: NE, NL, and MG. Investigation: NE, MC, MG, and AS. Methodology: NE, MG, and AS. Project administration: AS. Resources: AS, NE. Software: NE, MG, and ES. Supervision: MC and AS. Validation: NE and ES. Writing original draft: NE, ES, NL, and AS. Writing, review & editing: NE, ES, MC, MG, NL, and AS. All authors have read and agreed to the published version of the manuscript.
