# Supplementary material for: miRNome expression analysis in canine diffuse large B-cell lymphoma
Source: Front Oncol. 2023 Aug 30;13:1238613. doi: 10.3389/fonc.2023.1238613 (PMC10499539; doi:10.3389/fonc.2023.1238613)
Supplement: Supplementary file 1 [file Image_1.pdf]

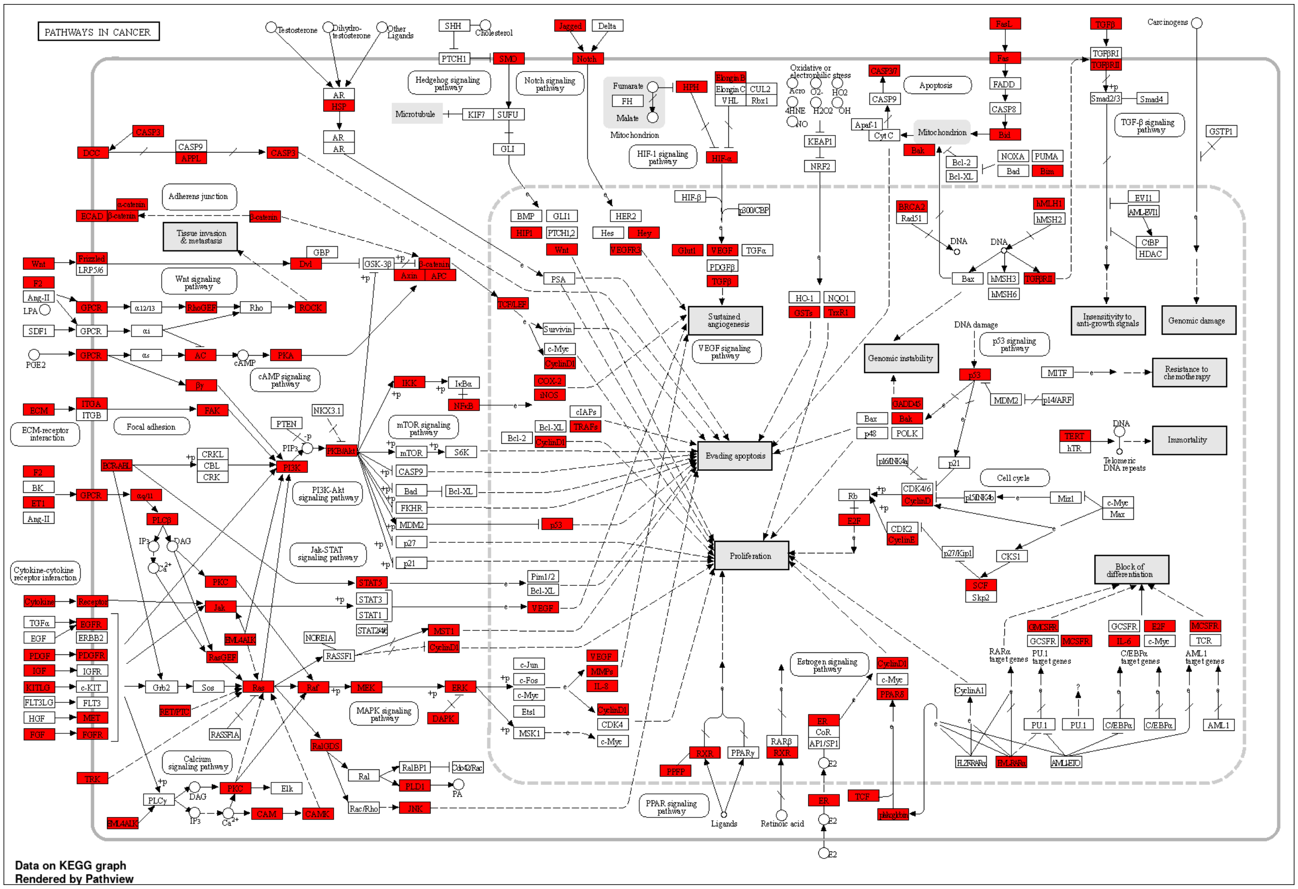

Figure S1: Kyoto Encyclopedia of Genes and Genomes (KEGG) diagram showing the predicted target genes of the the differentially expressed miRNAs (DEMs) enriched in the Pathways in cancer.

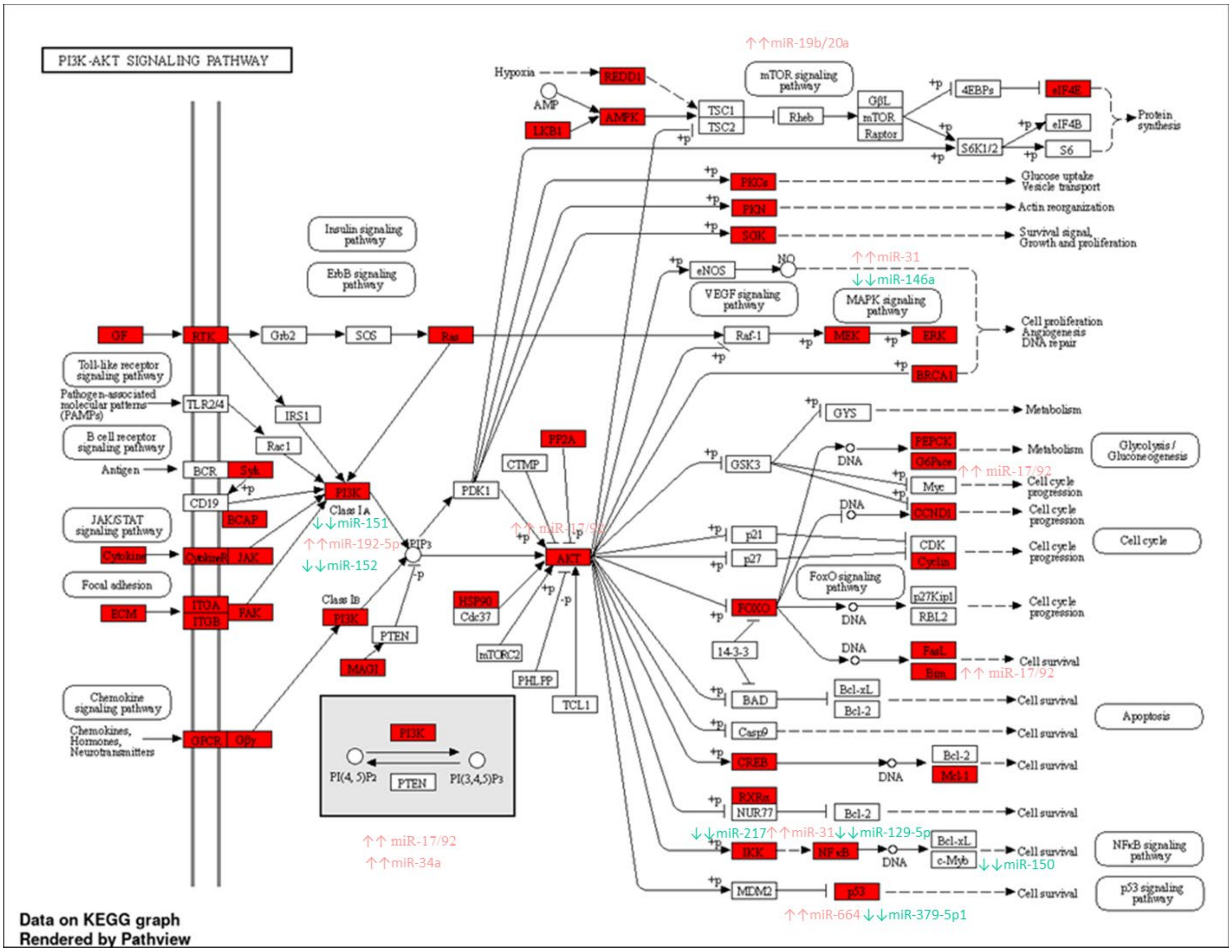

Figure S2: Kyoto Encyclopedia of Genes and Genomes (KEGG) diagram showing the predicted target genes of the differentially expressed miRNAs (DEMs) enriched in the PI3K-Akt pathway and the DEMs expression in each pathway, red represents overexpression while green represents downregulation.

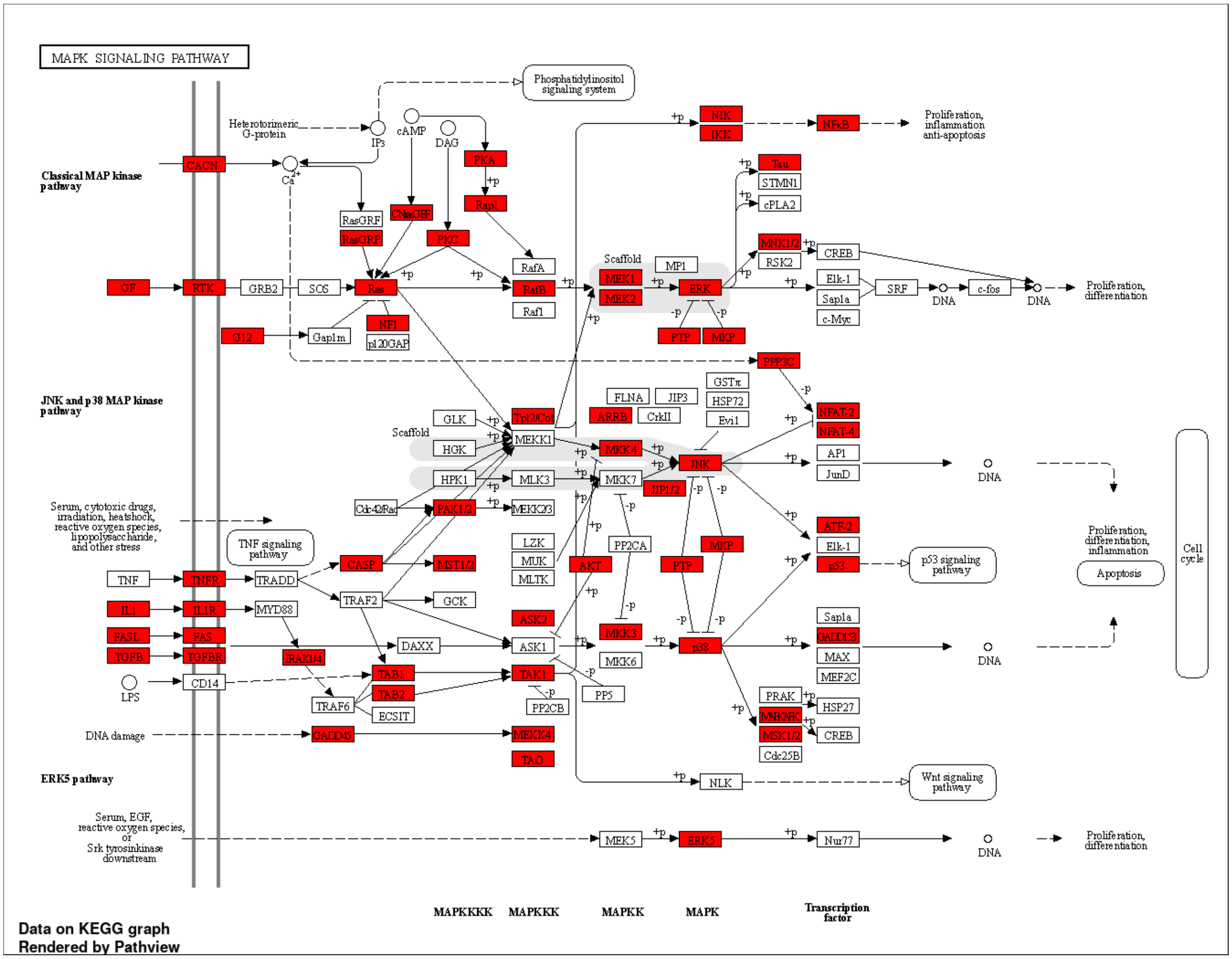

Figure S3: Kyoto Encyclopedia of Genes and Genomes (KEGG) diagram showing the predicted target genes of the differentially expressed miRNAs (DEMs) enriched in MAPK Pathways

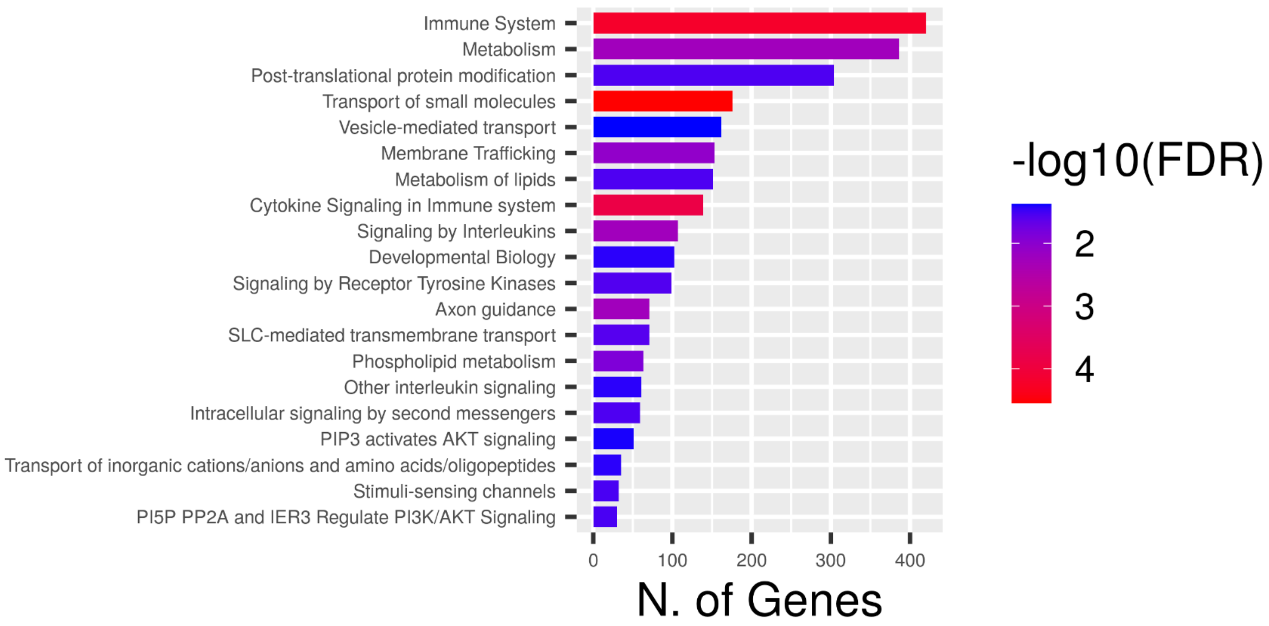

Figure S4: Reactome pathways annotation of the differentially expressed miRNAs (DEMs) targets in DLBCL. The name of each pathway is shown on the y-axis. Abscissa showed the number of genes enriched in each pathway. The bigger the fold enrichment is, the more significant the pathway. The color of the barplot represented negative log10 of the false discover rate (FDR) (red: high, blue: low).

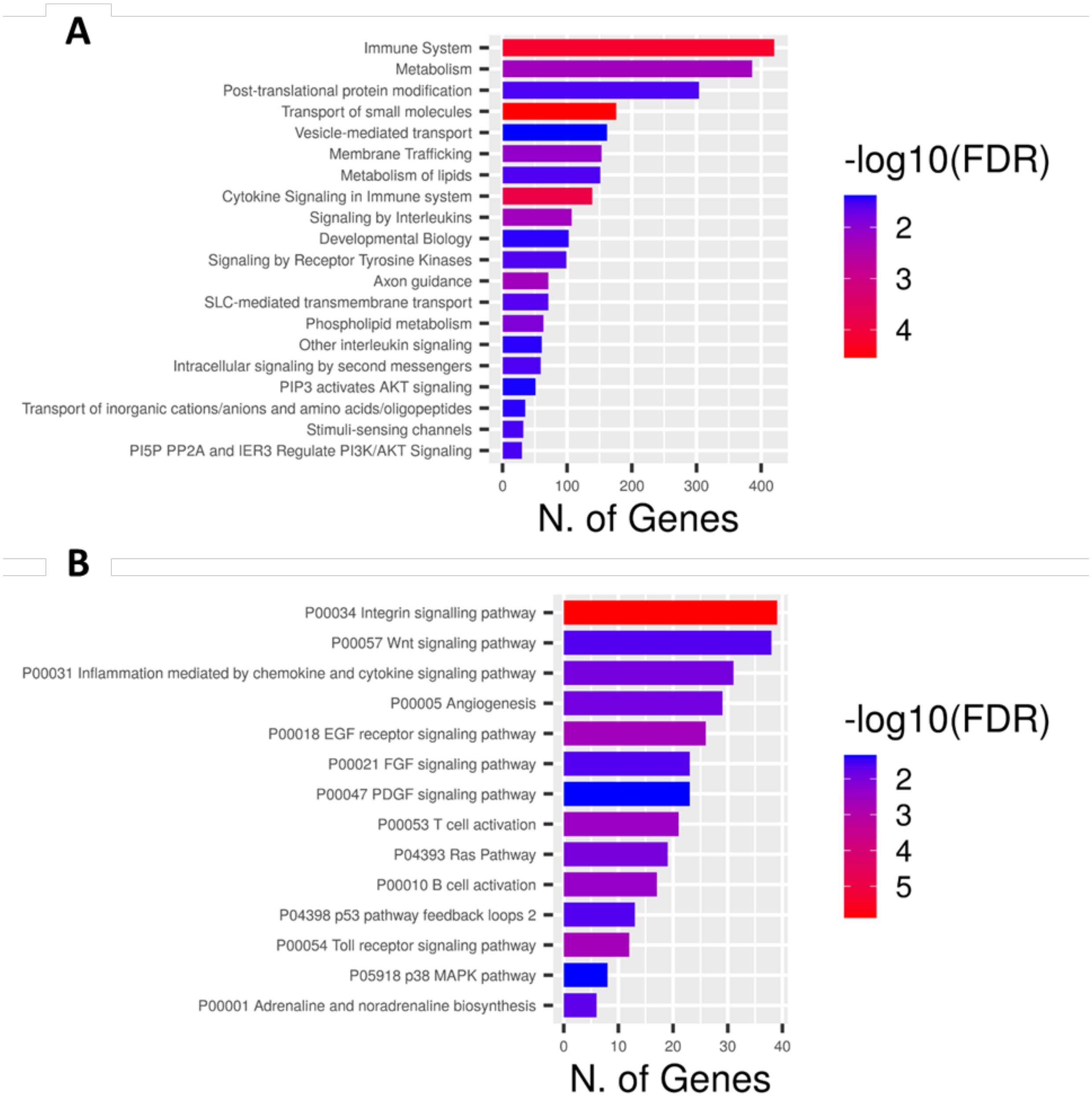

Figure S5: A) PANTHER pathways annotation of DEMs targets in DLBCL. The name of each pathway is shown on the y-axis. Abscissa showed the number of genes enriched in each pathway. The bigger the fold enrichment is, the more significant the pathway. The color of the barplot represented negative log10 of the false discover rate (FDR) (red: high, blue: low).

B) WikiPathways annotation of the differentially expressed miRNAs (DEMs) targets in DLBCL. The name of each pathway is shown on the y-axis. Abscissa showed the number of genes enriched in each pathway. The bigger the fold enrichment is, the more significant the pathway. The color of the barplot represented negative log10 of the false discover rate (FDR) (red: high, blue: low).
