# Supplementary material for: miRNome expression analysis in canine diffuse large B-cell lymphoma
Source: Front Oncol. 2023 Aug 30;13:1238613. doi: 10.3389/fonc.2023.1238613 (PMC10499539; doi:10.3389/fonc.2023.1238613)
Supplement: Supplementary file 2 [file Table_1.docx]

**Supplementary Table 1A**: Diffuse large B-cell lymphoma animals’ clinical information

| **Sample** | **Sex** | **Breed** | **History** | **Histopathology** | **Mitotic count** | **Tingible bodies** | **IHC** | **WHO stage at diagnosis** | **Cause of death** | **Progression-free survival (days)** | **Overall Survival (days)** |
| --- | --- | --- | --- | --- | --- | --- | --- | --- | --- | --- | --- |
| L1 | Castrated male | Mixed | lymphoma had been previously diagnosed. The patient has swelling of the neck. Chest radiographs were performed as well as a fine needle aspirate of lymph nodes. | Diffuse large B cell lymphoma, immunoblastic-centroblastic, intermediate/high grade | 10.2/hpf | yes | CD79a+CD3- | 5 | Lymphoma | 125 | 198 |
| L2 | Castrated male | Mixed | 1 month history of enlarged lymph nodes.  Suspect lymphoma | Diffuse, large cell lymphoma, intermediate grade | 7.1/hpf | yes | CD79a+Pax5+CD3- | 4 | Lost to follow-up | >176 | >176 |
| L3 | Neutered female | Golden | LN felt bigger/ generalized lymphadenopathy | Diffuse large B-cell lymphoma, immunoblastic type, intermediate grade | 7.4/hpf | no | CD79a+CD3- | 5 | Lymphoma | 65 | 183 |
| L4 | Intact male | Dogue de Bordeaux | diagnosis of lymphoma by fine needle aspirate. | Diffuse large cell lymphoma, intermediate grade | 5/hpf. | yes | CD79a+Pax5+CD3- | 4 | Lymphoma | 154 | 235 |
| L5 | Intact female | Basset hound | Previously diagnosed multicentric lymphoma. Upon presentation, she was in good spirits, and appeared to be in generally good condition apart from having numerous enlarged lymph nodes throughout her body. | Diffuse large cell lymphoma immunoblastic-centroblastic, intermediate grade | 4.1/hpf | Scant | Cd20+CD3- | 4 | Lymphoma | 16 | 100 |
| L6 | Castrated male | German Shepperd | Mandibular lymph node enlargement unresponsive to antibiotics. Biopsy prior to referral indicated aggressive nodal lymphoma | Diffuse, large cell lymphoma, centroblastic-immunoblastic, intermediate grade | 1.7/hpf | yes | CD79a+CD3- | 5 | Abdominal hemorrhage - etiology unspecified | 327 | 551 |

**Supplementary Table 1B**: Healthy animals’ demographic information

| **Symbol** | **Sex** | **Age (yr)** | **Breed** | **Weight (kg)** |
| --- | --- | --- | --- | --- |
| H2 | **Female** | **0.7** | **Hound X** | **25.0** |
| H3 | **Female** | **0.7** | **Hound X** | **23.6** |
| H4 | **Female** | **0.7** | **Hound X** | **25.0** |
| H5 | **Female** | **0.6** | **Hound X** | **16.4** |

**Supplementary Table 2:** Samples of RNA extraction information

| Sample IDs | total RNA ng/uL | 280/260 | 260/230 | Qubit miRNAs ng/uL |
| --- | --- | --- | --- | --- |
| Healthy 2 | 148 | 1.98 | 1.89 | 60.6 |
| Healthy 3 | 255 | 1.95 | 2.29 | 68.4 |
| Healthy 4 | 192 | 1.95 | 2.29 | 92.2 |
| Healthy 5 | 457 | 2.01 | 2.27 | 146 |
| DLBCL 1 | 246.1 | 1.99 | 2.26 | 134 |
| DLBCL 2 | 299.8 | 1.99 | 2.26 | 66 |
| DLBCL 3 | 58 | 1.97 | 2.17 | 16.5 |
| DLBCL 4 | 100.1 | 1.92 | 2.24 | 9.92 |
| DLBCL 5 | 79.6 | 1.98 | 2.11 | 17.4 |
| DLBCL 6 | 96.9 | 1.92 | 2.14 | 65.2 |

**Supplementary Table 3:** Raw data stat: Sample refers to sample identification. Reads relate to the statistics of the small RNA sequencing data. Bases stand for the sequence number times the length of the sequence in Giga base pairs. Error rate denotes to Sequencing error rate. Phred quality score (Q score) is logarithmically linked to base calling error. Q20 is the overall percentage of Phred values greater than 20 base. Q30 represents the overall percentage of Phred values greater than 30 bases. G.C. content refers to the G and C bases within the overall base.

| Sample | Reads | Bases | Error rate | Q20 | Q30 | GC content |
| --- | --- | --- | --- | --- | --- | --- |
| DLBCL1 | 5.4E+07 | 2.713G | 0.01% | 99.40% | 97.91% | 50.17% |
| DLBCL2 | 5.4E+07 | 2.707G | 0.01% | 99.53% | 98.22% | 49.90% |
| DLBCL3 | 6.2E+07 | 3.115G | 0.01% | 99.54% | 98.30% | 49.64% |
| DLBCL4 | 5.2E+07 | 2.605G | 0.01% | 98.75% | 95.95% | 49.08% |
| DLBCL5 | 5.1E+07 | 2.532G | 0.01% | 99.50% | 98.08% | 49.47% |
| DLBCL6 | 5E+07 | 2.524G | 0.01% | 98.52% | 95.19% | 49.63% |
| Healthy2 | 5E+07 | 2.525G | 0.01% | 98.85% | 96.46% | 48.51% |
| Healthy3 | 5E+07 | 2.498G | 0.01% | 98.73% | 96.07% | 48.64% |
| Healthy4 | 5E+07 | 2.509G | 0.01% | 98.80% | 96.19% | 48.72% |
| Healthy5 | 5.1E+07 | 2.549G | 0.01% | 98.89% | 96.50% | 48.42% |

**Supplementary Table 4:** The data cleaning statistics of each sample. Total reads refer to total sequenced reads. N% > 10% represents the percentage of reads with poly-N. The percentage of low-quality reads equals 0%. The percentage of reads with '5'adapter contaminations is less than 0.02%. The percentage of reads without a '3' adapter or insert is less than 8%. The percentage of reads with Ploy A/T/G/C tails is less than 3%. Total clean reads as a percentage of raw reads are more than 89%.

| Sample | Total reads | N% > 10% | Low quality | 5_adapter contamine | 3_adapter_null or insert null | With ploy A/T/G/C | Clean reads |
| --- | --- | --- | --- | --- | --- | --- | --- |
| DLBCL1 | 54260466 (100.00%) | 1226 (0.00%) | 0 (0.00%) | 6546 (0.01%) | 4383713 (8.08%) | 1496071 (2.76%) | 48372910 (89.15%) |
| DLBCL2 | 54146156 (100.00%) | 1185 (0.00%) | 0 (0.00%) | 2584 (0.00%) | 659833 (1.22%) | 244775 (0.45%) | 53237779 (98.32%) |
| DLBCL3 | 62294056 (100.00%) | 1410 (0.00%) | 0 (0.00%) | 3572 (0.01%) | 699057 (1.12%) | 395878 (0.64%) | 61194139 (98.23%) |
| DLBCL4 | 52107088 (100.00%) | 761 (0.00%) | 0 (0.00%) | 8010 (0.02%) | 2440768 (4.68%) | 760642 (1.46%) | 48896907 (93.84%) |
| DLBCL5 | 50642538 (100.00%) | 1124 (0.00%) | 0 (0.00%) | 4233 (0.01%) | 1199612 (2.37%) | 278982 (0.55%) | 49158587 (97.07%) |
| DLBCL6 | 50483967 (100.00%) | 1081 (0.00%) | 0 (0.00%) | 6028 (0.01%) | 2178306 (4.31%) | 116769 (0.23%) | 48181783 (95.44%) |
| Healthy2 | 50491187 (100.00%) | 713 (0.00%) | 0 (0.00%) | 871 (0.00%) | 285191 (0.56%) | 72114 (0.14%) | 50132298 (99.29%) |
| Healthy3 | 49952302 (100.00%) | 1129 (0.00%) | 0 (0.00%) | 1024 (0.00%) | 417821 (0.84%) | 92268 (0.18%) | 49440060 (98.97%) |
| Healthy4 | 50189807 (100.00%) | 1131 (0.00%) | 0 (0.00%) | 1527 (0.00%) | 704940 (1.40%) | 21418 (0.04%) | 49460791 (98.55%) |
| Healthy5 | 50977527 (100.00%) | 1169 (0.00%) | 0 (0.00%) | 1575 (0.00%) | 651615 (1.28%) | 63892 (0.13%) | 50259276 (98.59%) |

**Supplementary Table 5:** The reference mapping statistics. Total sRNA means the number of total small RNAs after the length filter. Mapped sRNA refers to the number of sRNAs mapped to the genome, and its percentage "+" Mapped sRNA refers to the number of mapped sRNAs in the same direction as the genome and its percentage while "- "Mapped sRNA refers to the number of mapped sRNAs in the opposite direction the genome its percentage.

| Sample | Total sRNA | Mapped sRNA | “+” Mapped sRNA | “-“ Mapped sRNA |
| --- | --- | --- | --- | --- |
| DLBCL1 | 43531152 (100.00%) | 29054623 (66.74%) | 18481017 (42.45%) | 10573606 (24.29%) |
| DLBCL3 | 58084783 (100.00%) | 41489325 (71.43%) | 20833051 (35.87%) | 20656274 (35.56%) |
| DLBCL2 | 51507163 (100.00%) | 35669186 (69.25%) | 20559742 (39.92%) | 15109444 (29.33%) |
| Healthy2 | 49417176 (100.00%) | 38835606 (78.59%) | 7733564 (15.65%) | 31102042 (62.94%) |
| DLBCL4 | 45000620 (100.00%) | 32975154 (73.28%) | 19138638 (42.53%) | 13836516 (30.75%) |
| DLBCL5 | 45902459 (100.00%) | 34028229 (74.13%) | 16809779 (36.62%) | 17218450 (37.51%) |
| Healthy4 | 47979259 (100.00%) | 36384742 (75.83%) | 10846718 (22.61%) | 25538024 (53.23%) |
| Healthy5 | 47983296 (100.00%) | 36055373 (75.14%) | 12615333 (26.29%) | 23440040 (48.85%) |
| DLBCL6 | 38144581 (100.00%) | 28753069 (75.38%) | 15248629 (39.98%) | 13504440 (35.40%) |
| Healthy3 | 48328650 (100.00%) | 37709838 (78.03%) | 12656444 (26.19%) | 25053394 (51.84%) |

**Supplementary Table 6:** DLBCL vs. Healthy expression analysis results.

| sRNA | DLBCL_readcount | Healthy_readcount | log2FoldChange | *P-value* | *P-adj* | Significant |
| --- | --- | --- | --- | --- | --- | --- |
| cfa-let-7a | 630352 | 473963 | 0.38508 | 0.37109 | 0.59924 | FALSE |
| cfa-let-7b | 56808.2 | 66977.9 | -0.2318 | 0.39397 | 0.6269 | FALSE |
| cfa-let-7c | 53433 | 67094.3 | -0.3075 | 0.47605 | 0.6908 | FALSE |
| cfa-let-7d | 3713.27 | 2752.24 | 0.38806 | 0.46552 | 0.6908 | FALSE |
| cfa-let-7e | 8173.28 | 9930.29 | -0.2706 | 0.41228 | 0.64659 | FALSE |
| cfa-let-7f | 2507256 | 2516277 | -0.0046 | 0.99322 | 0.99503 | FALSE |
| cfa-let-7g | 1655712 | 1554619 | 0.08518 | 0.84205 | 0.91784 | FALSE |
| cfa-miR-1 | 4285.52 | 1168.77 | 1.4504 | NA | NA | NA |
| cfa-miR-101 | 103185 | 102767 | 0.00568 | 0.98471 | 0.99503 | FALSE |
| cfa-miR-105a | 215.669 | 179.619 | 0.23397 | NA | NA | NA |
| cfa-miR-105b | 10.271 | 10.0239 | 0.03445 | 0.9669 | NA | NA |
| cfa-miR-106a | 188.204 | 40.5442 | 1.9197 | 0.00108 | 0.0138 | TRUE |
| cfa-miR-106b | 16036.2 | 7873.09 | 0.9414 | 0.05175 | 0.1635 | FALSE |
| cfa-miR-107 | 3771.31 | 2239.18 | 0.7113 | 0.07395 | 0.20152 | FALSE |
| cfa-miR-10a | 85156.9 | 227774 | -1.2564 | 0.02585 | 0.09897 | FALSE |
| cfa-miR-10b | 1000155 | 783106 | 0.29941 | NA | NA | NA |
| cfa-miR-1185 | 12.0057 | 19.5697 | -0.4666 | 0.5644 | 0.75024 | FALSE |
| cfa-miR-1199-3p | 0.27559 | 0 | 0.12677 | 0.74125 | NA | NA |
| cfa-miR-122 | 186.483 | 58.5601 | 1.3547 | 0.04943 | 0.15945 | FALSE |
| cfa-miR-124 | 6.06312 | 6.58087 | -0.0833 | 0.92404 | NA | NA |
| cfa-miR-125a | 9865.64 | 20464.9 | -1.0011 | 0.00851 | 0.05013 | FALSE |
| cfa-miR-125b | 17774 | 47790.3 | -1.2856 | 0.01491 | 0.06815 | FALSE |
| cfa-miR-126 | 3929.94 | 8420.62 | -0.9364 | 0.13596 | 0.29057 | FALSE |
| cfa-miR-127 | 1803.67 | 5411.51 | -1.3243 | 0.04352 | 0.14595 | FALSE |
| cfa-miR-1271 | 33047.2 | 16437.2 | 0.91188 | NA | NA | NA |
| cfa-miR-128 | 157023 | 35587.5 | 1.8893 | 0.0008 | 0.01161 | TRUE |
| cfa-miR-129 | 1613.74 | 1425.79 | 0.14093 | 0.84411 | 0.91784 | FALSE |
| cfa-miR-1296 | 189.414 | 67.3439 | 1.3064 | NA | NA | NA |
| cfa-miR-1301 | 1145.42 | 709.556 | 0.61615 | 0.25819 | 0.43974 | FALSE |
| cfa-miR-1306 | 918.987 | 483.017 | 0.84847 | 0.08407 | 0.21634 | FALSE |
| cfa-miR-1307 | 4357.25 | 1297.73 | 1.4567 | NA | NA | NA |
| cfa-miR-130a | 202.075 | 265.029 | -0.3415 | 0.55124 | 0.74472 | FALSE |
| cfa-miR-130b | 564.843 | 305.813 | 0.83045 | 0.04974 | 0.15945 | FALSE |
| cfa-miR-132 | 563.752 | 636.058 | -0.1603 | 0.73868 | 0.85999 | FALSE |
| cfa-miR-133b | 13.5638 | 2.49684 | 1.4772 | 0.0814 | NA | NA |
| cfa-miR-133c | 264.239 | 77.8986 | 1.524 | 0.01067 | 0.05812 | FALSE |
| cfa-miR-134 | 26.0638 | 35.5724 | -0.3361 | 0.64146 | 0.80831 | FALSE |
| cfa-miR-1343 | 1512.12 | 464.048 | 1.5108 | 0.00649 | 0.04164 | TRUE |
| cfa-miR-135a-3p | 0.29269 | 33.5624 | -4.283 | NA | NA | NA |
| cfa-miR-135a-5p | 49.5699 | 275.619 | -1.7757 | 0.02588 | 0.09897 | FALSE |
| cfa-miR-135b | 66.8877 | 9.81667 | 1.9406 | 0.01435 | 0.06815 | FALSE |
| cfa-miR-136 | 112.195 | 302.53 | -0.9897 | 0.22523 | 0.40578 | FALSE |
| cfa-miR-137 | 0.83677 | 0 | 0.31419 | NA | NA | NA |
| cfa-miR-138a | 420.552 | 667.47 | -0.6012 | 0.25127 | 0.43473 | FALSE |
| cfa-miR-138b | 25.2259 | 9.30745 | 1.0942 | 0.14195 | 0.29754 | FALSE |
| cfa-miR-139 | 11.7146 | 45.9034 | -1.6722 | 0.00633 | 0.04164 | TRUE |
| cfa-miR-140 | 122748 | 111521 | 0.12955 | 0.76346 | 0.87139 | FALSE |
| cfa-miR-141 | 184.297 | 197.012 | -0.0919 | 0.85048 | 0.91784 | FALSE |
| cfa-miR-142 | 248224 | 210940 | 0.227 | 0.47218 | 0.6908 | FALSE |
| cfa-miR-143 | 1429588 | 1553246 | -0.1101 | 0.81748 | 0.90462 | FALSE |
| cfa-miR-144 | 512.889 | 74.9671 | 2.1051 | 0.00444 | 0.03338 | TRUE |
| cfa-miR-145 | 25506.7 | 31554.2 | -0.2768 | 0.59752 | 0.77077 | FALSE |
| cfa-miR-1468 | 53.332 | 157.788 | -1.3887 | 0.01392 | 0.06815 | FALSE |
| cfa-miR-146a | 166696 | 455270 | -1.3814 | 0.00022 | 0.00483 | TRUE |
| cfa-miR-146b | 34275.5 | 18144.2 | 0.82283 | 0.12384 | 0.28716 | FALSE |
| cfa-miR-147 | 151.28 | 105.31 | 0.44062 | 0.49121 | 0.69891 | FALSE |
| cfa-miR-148a | 1.4E+07 | 4.2E+07 | -1.3777 | 0.01365 | 0.06815 | FALSE |
| cfa-miR-148b | 70592.8 | 46907.5 | 0.54853 | 0.22106 | 0.40159 | FALSE |
| cfa-miR-149 | 216.934 | 197.295 | 0.12573 | 0.78951 | 0.88599 | FALSE |
| cfa-miR-150 | 48674.6 | 352411 | -2.578 | 9.30E-07 | 6.76E-05 | TRUE |
| cfa-miR-151 | 9827.9 | 25999.4 | -1.3413 | 0.00022 | 0.00483 | TRUE |
| cfa-miR-152 | 24067.9 | 67919.2 | -1.3677 | 0.00582 | 0.03964 | TRUE |
| cfa-miR-153 | 223.621 | 211.284 | 0.07074 | 0.90376 | 0.95641 | FALSE |
| cfa-miR-155 | 351729 | 252002 | 0.44043 | 0.36775 | 0.59828 | FALSE |
| cfa-miR-15a | 1208.37 | 1423.04 | -0.2127 | 0.68398 | 0.83275 | FALSE |
| cfa-miR-15b | 16624.4 | 11036 | 0.55767 | 0.16951 | 0.33593 | FALSE |
| cfa-miR-16 | 54329.8 | 63530.7 | -0.2132 | 0.59733 | 0.77077 | FALSE |
| cfa-miR-17 | 683.962 | 221.184 | 1.4681 | 0.00477 | 0.03465 | TRUE |
| cfa-miR-181a | 44655.5 | 122664 | -1.3079 | 0.01487 | 0.06815 | FALSE |
| cfa-miR-181b | 18597 | 23284.3 | -0.286 | 0.61519 | 0.78427 | FALSE |
| cfa-miR-181c | 163.384 | 384.639 | -1.0201 | 0.12791 | 0.28716 | FALSE |
| cfa-miR-181d | 1458.64 | 1984.19 | -0.4031 | 0.42803 | 0.6665 | FALSE |
| cfa-miR-182 | 127055 | 10410.6 | 2.4997 | NA | NA | NA |
| cfa-miR-183 | 58521.8 | 3842.63 | 2.6074 | NA | NA | NA |
| cfa-miR-1835 | 248.191 | 175.181 | 0.47718 | 0.20058 | 0.37131 | FALSE |
| cfa-miR-1836 | 376.575 | 127.54 | 1.3574 | 0.02151 | 0.08524 | FALSE |
| cfa-miR-1837 | 2.09781 | 12.3225 | -1.329 | NA | NA | NA |
| cfa-miR-1838 | 126.432 | 178.741 | -0.474 | 0.23081 | 0.40908 | FALSE |
| cfa-miR-1839 | 36608.9 | 7351.29 | 2.0456 | 0.00027 | 0.00527 | TRUE |
| cfa-miR-184 | 313.129 | 46.3259 | 1.758 | NA | NA | NA |
| cfa-miR-1840 | 243.331 | 51.6179 | 1.8649 | 0.00415 | 0.03338 | TRUE |
| cfa-miR-1841 | 252.174 | 90.4515 | 1.2277 | NA | NA | NA |
| cfa-miR-1842 | 288.111 | 106.067 | 1.3437 | 0.00162 | 0.01849 | TRUE |
| cfa-miR-1843 | 548.421 | 220.731 | 1.107 | NA | NA | NA |
| cfa-miR-1844 | 1588.7 | 564.798 | 1.2619 | NA | NA | NA |
| cfa-miR-185 | 24063 | 13997.7 | 0.70273 | NA | NA | NA |
| cfa-miR-186 | 268660 | 172849 | 0.60268 | 0.12555 | 0.28716 | FALSE |
| cfa-miR-187 | 1224.63 | 526.192 | 0.91118 | 0.23066 | 0.40908 | FALSE |
| cfa-miR-188 | 224.46 | 99.2808 | 1.0379 | 0.06401 | 0.18123 | FALSE |
| cfa-miR-18a | 2602.56 | 734.334 | 1.61 | 0.00431 | 0.03338 | TRUE |
| cfa-miR-18b | 131.978 | 49.5843 | 1.1827 | 0.0673 | 0.1881 | FALSE |
| cfa-miR-190a | 64.8035 | 65.2179 | -0.0057 | 0.99262 | 0.99503 | FALSE |
| cfa-miR-190b | 610.054 | 314.213 | 0.88294 | 0.05766 | 0.17205 | FALSE |
| cfa-miR-191 | 498784 | 259968 | 0.89527 | 0.01688 | 0.07216 | FALSE |
| cfa-miR-192 | 87123.4 | 26700.8 | 1.5841 | 0.00046 | 0.00773 | TRUE |
| cfa-miR-193a | 1119.34 | 1344.71 | -0.2481 | 0.55825 | 0.74661 | FALSE |
| cfa-miR-193b | 20.515 | 65.8183 | -1.3955 | 0.03423 | 0.12234 | FALSE |
| cfa-miR-194 | 10566.4 | 4574.04 | 1.1301 | 0.00874 | 0.05014 | FALSE |
| cfa-miR-195 | 10214.7 | 20528.7 | -0.9508 | 0.0188 | 0.0788 | FALSE |
| cfa-miR-196a | 2152.46 | 2881.8 | -0.3113 | 0.68759 | 0.83275 | FALSE |
| cfa-miR-196b | 1228.18 | 4670.16 | -1.5448 | 0.02876 | 0.10808 | FALSE |
| cfa-miR-197 | 10540.4 | 10857 | -0.0372 | 0.94981 | 0.9767 | FALSE |
| cfa-miR-199 | 71815.6 | 139211 | -0.8545 | 0.11471 | 0.26888 | FALSE |
| cfa-miR-19a | 1342.66 | 329.582 | 1.6157 | 0.02104 | 0.08495 | FALSE |
| cfa-miR-19b | 13709.7 | 3923.9 | 1.611 | 0.00296 | 0.02688 | TRUE |
| cfa-miR-200a | 145.356 | 3.66624 | 2.3926 | NA | NA | NA |
| cfa-miR-200b | 0.46683 | 0 | 0.19671 | 0.67278 | NA | NA |
| cfa-miR-200c | 1513.65 | 607.621 | 1.0857 | NA | NA | NA |
| cfa-miR-202 | 9.11819 | 7.66452 | 0.14997 | NA | NA | NA |
| cfa-miR-203 | 596.554 | 437.504 | 0.38226 | 0.53784 | 0.73861 | FALSE |
| cfa-miR-204 | 357.82 | 937.219 | -1.2246 | 0.03205 | 0.11841 | FALSE |
| cfa-miR-205 | 4.23613 | 2.45979 | 0.39038 | 0.65754 | NA | NA |
| cfa-miR-206 | 656.078 | 411.08 | 0.48408 | 0.54034 | 0.73861 | FALSE |
| cfa-miR-208a | 0.16735 | 0.88479 | -0.3835 | 0.56397 | NA | NA |
| cfa-miR-208b | 7.04785 | 3.85424 | 0.58694 | 0.47282 | NA | NA |
| cfa-miR-20a | 133341 | 33819.2 | 1.7829 | 0.00066 | 0.0102 | TRUE |
| cfa-miR-20b | 7767.14 | 1823.4 | 1.8254 | 0.0017 | 0.01849 | TRUE |
| cfa-miR-21 | 6271416 | 5714160 | 0.12666 | 0.75535 | 0.86666 | FALSE |
| cfa-miR-210 | 742.711 | 934.835 | -0.3043 | 0.53087 | 0.73714 | FALSE |
| cfa-miR-211 | 3.4834 | 1.54501 | 0.39274 | 0.64069 | NA | NA |
| cfa-miR-2114 | 1.99043 | 2.32102 | -0.0725 | 0.93382 | NA | NA |
| cfa-miR-212 | 105.67 | 56.6757 | 0.74543 | NA | NA | NA |
| cfa-miR-214 | 2144.98 | 3645.17 | -0.6955 | 0.16947 | 0.33593 | FALSE |
| cfa-miR-215 | 686.937 | 551.549 | 0.29939 | 0.44601 | 0.68296 | FALSE |
| cfa-miR-216a | 469.03 | 2730.65 | -1.8848 | 0.01516 | 0.06815 | FALSE |
| cfa-miR-216b | 6698.96 | 49167.5 | -2.325 | 0.00087 | 0.01192 | TRUE |
| cfa-miR-217 | 1693.52 | 15019 | -2.3583 | 0.00217 | 0.02155 | TRUE |
| cfa-miR-218 | 16718.2 | 15693.7 | 0.08193 | 0.87787 | 0.93837 | FALSE |
| cfa-miR-219-3p | 6.1424 | 1.15114 | 1.0178 | 0.24624 | NA | NA |
| cfa-miR-219-5p | 54.9385 | 29.4242 | 0.80923 | 0.101 | 0.24656 | FALSE |
| cfa-miR-22 | 8124.18 | 8658.69 | -0.0882 | 0.79658 | 0.88599 | FALSE |
| cfa-miR-221 | 11459.6 | 11483.5 | -0.0028 | 0.99503 | 0.99503 | FALSE |
| cfa-miR-222 | 52699.1 | 21998.6 | 1.0849 | 0.07379 | 0.20152 | FALSE |
| cfa-miR-223 | 1358.68 | 1231.61 | 0.12054 | 0.84747 | 0.91784 | FALSE |
| cfa-miR-224 | 1212.78 | 745.16 | 0.59168 | 0.35631 | 0.58403 | FALSE |
| cfa-miR-2387 | 0.16735 | 0 | 0.08067 | 0.79581 | NA | NA |
| cfa-miR-23a | 3611.39 | 7892.2 | -1.0096 | 0.06195 | 0.17864 | FALSE |
| cfa-miR-23b | 1766.98 | 3294.14 | -0.7935 | 0.16147 | 0.32593 | FALSE |
| cfa-miR-24 | 146753 | 136997 | 0.0948 | 0.79478 | 0.88599 | FALSE |
| cfa-miR-2483 | 2.10649 | 3.46041 | -0.257 | 0.7637 | NA | NA |
| cfa-miR-25 | 508144 | 200954 | 1.1944 | NA | NA | NA |
| cfa-miR-26a | 846971 | 1220194 | -0.5107 | 0.09207 | 0.23338 | FALSE |
| cfa-miR-26b | 238899 | 243203 | -0.0246 | 0.94696 | 0.9767 | FALSE |
| cfa-miR-27a | 93271.7 | 116104 | -0.2927 | 0.5243 | 0.73267 | FALSE |
| cfa-miR-27b | 313178 | 259668 | 0.24988 | 0.59122 | 0.77077 | FALSE |
| cfa-miR-28 | 40245.3 | 46610.1 | -0.1863 | 0.74542 | 0.85999 | FALSE |
| cfa-miR-299 | 6.64956 | 32.323 | -1.6563 | 0.03391 | 0.12234 | FALSE |
| cfa-miR-29a | 72622.6 | 90489 | -0.299 | 0.46759 | 0.6908 | FALSE |
| cfa-miR-29b | 18526 | 11564.9 | 0.63243 | 0.15778 | 0.32145 | FALSE |
| cfa-miR-29c | 3784.53 | 970.029 | 1.7318 | 0.00216 | 0.02155 | TRUE |
| cfa-miR-300 | 1.04542 | 2.90897 | -0.5094 | 0.54462 | NA | NA |
| cfa-miR-301a | 26.0953 | 20.645 | 0.26694 | 0.69933 | 0.84229 | FALSE |
| cfa-miR-301b | 143.421 | 69.6597 | 0.8991 | 0.12909 | 0.28716 | FALSE |
| cfa-miR-302a | 10.8886 | 25.1653 | -0.9627 | 0.17936 | 0.34912 | FALSE |
| cfa-miR-302b | 0.65242 | 0 | 0.25461 | 0.6235 | NA | NA |
| cfa-miR-30a | 261996 | 430309 | -0.6666 | 0.13547 | 0.29057 | FALSE |
| cfa-miR-30b | 35447.5 | 25890.1 | 0.40641 | 0.448 | 0.68296 | FALSE |
| cfa-miR-30c | 214833 | 189066 | 0.17497 | 0.65068 | 0.81521 | FALSE |
| cfa-miR-30d | 1225417 | 472152 | 1.2603 | 0.00966 | 0.05401 | FALSE |
| cfa-miR-30e | 77290.8 | 56538.9 | 0.3955 | 0.49372 | 0.69891 | FALSE |
| cfa-miR-31 | 13184.4 | 1048.43 | 3.2476 | 2.64E-09 | 2.88E-07 | TRUE |
| cfa-miR-32 | 3350.94 | 1999.13 | 0.63181 | 0.31632 | 0.5224 | FALSE |
| cfa-miR-320 | 24023.8 | 27209.3 | -0.1646 | 0.73597 | 0.85999 | FALSE |
| cfa-miR-323 | 26.958 | 86.4978 | -1.2567 | 0.10179 | 0.24656 | FALSE |
| cfa-miR-324 | 251.627 | 232.215 | 0.1084 | 0.78751 | 0.88599 | FALSE |
| cfa-miR-325 | 0 | 0.31509 | -0.1499 | 0.66561 | NA | NA |
| cfa-miR-326 | 216.785 | 311.357 | -0.4689 | 0.37775 | 0.60551 | FALSE |
| cfa-miR-328 | 655.565 | 315.777 | 0.97185 | 0.03842 | 0.13086 | FALSE |
| cfa-miR-329b | 11.7901 | 15.7899 | -0.3057 | 0.67988 | NA | NA |
| cfa-miR-330 | 14213.3 | 5701.14 | 1.1066 | NA | NA | NA |
| cfa-miR-331 | 1155.29 | 1160.36 | -0.0056 | 0.99024 | 0.99503 | FALSE |
| cfa-miR-335 | 51.2159 | 51.9514 | -0.0123 | 0.98396 | 0.99503 | FALSE |
| cfa-miR-338 | 172.48 | 315.07 | -0.7305 | 0.25652 | 0.43974 | FALSE |
| cfa-miR-339 | 10624.7 | 12271.8 | -0.1879 | 0.71725 | 0.85912 | FALSE |
| cfa-miR-33a | 138.26 | 62.5902 | 0.90287 | 0.20503 | 0.3756 | FALSE |
| cfa-miR-33b | 1.63862 | 0 | 1.001 | 0.21571 | NA | NA |
| cfa-miR-340 | 81293.3 | 90009.6 | -0.1386 | 0.73387 | 0.85999 | FALSE |
| cfa-miR-342 | 3577.8 | 5846.39 | -0.6735 | 0.07868 | 0.20917 | FALSE |
| cfa-miR-345 | 68.6048 | 59.3343 | 0.18463 | 0.68233 | 0.83275 | FALSE |
| cfa-miR-346 | 0 | 0.75726 | -0.3203 | 0.5101 | NA | NA |
| cfa-miR-34a | 13257.1 | 820.575 | 3.1991 | 3.72E-06 | 0.0002 | TRUE |
| cfa-miR-34b | 10.9617 | 3.04824 | 0.91845 | NA | NA | NA |
| cfa-miR-34c | 1902.1 | 977.546 | 0.69183 | NA | NA | NA |
| cfa-miR-350 | 913.928 | 1072.32 | -0.2027 | 0.72258 | 0.85999 | FALSE |
| cfa-miR-361 | 2150.43 | 2424.76 | -0.1675 | 0.59656 | 0.77077 | FALSE |
| cfa-miR-362 | 3565.26 | 2374.11 | 0.56156 | 0.11432 | 0.26888 | FALSE |
| cfa-miR-363 | 428689 | 91027 | 2.0299 | 5.98E-05 | 0.00217 | TRUE |
| cfa-miR-365 | 796.869 | 1329.91 | -0.681 | 0.15138 | 0.3143 | FALSE |
| cfa-miR-369 | 0.16735 | 1.18162 | -0.3768 | 0.54287 | NA | NA |
| cfa-miR-370 | 272.95 | 354.529 | -0.3155 | 0.62345 | 0.79019 | FALSE |
| cfa-miR-371 | 46.1222 | 24.1356 | 0.75524 | NA | NA | NA |
| cfa-miR-374a | 22998.2 | 19039 | 0.23876 | 0.68058 | 0.83275 | FALSE |
| cfa-miR-374b | 16830.5 | 17212.3 | -0.0298 | 0.94971 | 0.9767 | FALSE |
| cfa-miR-375 | 730.917 | 135.254 | 1.4799 | NA | NA | NA |
| cfa-miR-376a | 8.89318 | 22.9213 | -0.9321 | 0.25115 | 0.43473 | FALSE |
| cfa-miR-376b | 1.42924 | 3.24754 | -0.5063 | 0.56275 | NA | NA |
| cfa-miR-376c | 5.12742 | 11.4465 | -0.7117 | 0.40253 | NA | NA |
| cfa-miR-377 | 0.74242 | 0.31509 | 0.19884 | 0.77156 | NA | NA |
| cfa-miR-378 | 297908 | 80917.2 | 1.7248 | 0.00035 | 0.00643 | TRUE |
| cfa-miR-379 | 2457.48 | 9632.74 | -1.7143 | 0.004 | 0.03338 | TRUE |
| cfa-miR-380 | 28.1595 | 110.527 | -1.4753 | 0.05348 | 0.16654 | FALSE |
| cfa-miR-381 | 8276.34 | 24074.7 | -1.3116 | 0.03715 | 0.13064 | FALSE |
| cfa-miR-382 | 61.2289 | 244.538 | -1.5073 | 0.04722 | 0.15597 | FALSE |
| cfa-miR-383 | 90.7716 | 138.727 | -0.4623 | 0.5421 | 0.73861 | FALSE |
| cfa-miR-384 | 0 | 0.31509 | -0.1499 | 0.66561 | NA | NA |
| cfa-miR-3958 | 72.6858 | 168.544 | -1.0298 | 0.09874 | 0.2446 | FALSE |
| cfa-miR-410 | 7.20982 | 23.6353 | -1.1617 | 0.15342 | 0.31553 | FALSE |
| cfa-miR-411 | 1612.6 | 4598.2 | -1.2493 | 0.06228 | 0.17864 | FALSE |
| cfa-miR-421 | 466.434 | 272.567 | 0.69476 | 0.19523 | 0.3669 | FALSE |
| cfa-miR-423a | 89295.1 | 36871.8 | 1.163 | 0.01969 | 0.08101 | FALSE |
| cfa-miR-424 | 37.0323 | 19.6554 | 0.82514 | 0.13507 | 0.29057 | FALSE |
| cfa-miR-425 | 164060 | 53659.5 | 1.4739 | 0.00269 | 0.02553 | TRUE |
| cfa-miR-429 | 7.0562 | 0 | 2.1541 | NA | NA | NA |
| cfa-miR-432 | 47.7784 | 180.586 | -1.6063 | 0.01275 | 0.0662 | FALSE |
| cfa-miR-433 | 14.5864 | 26.5627 | -0.6976 | 0.29382 | 0.49654 | FALSE |
| cfa-miR-448 | 0.16735 | 0 | 0.08067 | 0.79581 | NA | NA |
| cfa-miR-449a | 128.56 | 15.4203 | 1.8852 | NA | NA | NA |
| cfa-miR-449b | 0.27559 | 0 | 0.12677 | 0.74125 | NA | NA |
| cfa-miR-450a | 2315.42 | 2243.16 | 0.04083 | 0.94068 | 0.9767 | FALSE |
| cfa-miR-450b | 5569.82 | 2212.95 | 1.1471 | 0.05814 | 0.17205 | FALSE |
| cfa-miR-451 | 875605 | 37424.5 | 4.1068 | 1.27E-15 | 2.76E-13 | TRUE |
| cfa-miR-452 | 1795.6 | 1527.35 | 0.20339 | 0.73154 | 0.85999 | FALSE |
| cfa-miR-454 | 757.556 | 562.711 | 0.40479 | 0.3065 | 0.51398 | FALSE |
| cfa-miR-455 | 13987.7 | 17132 | -0.2673 | 0.58847 | 0.77077 | FALSE |
| cfa-miR-483 | 0.33471 | 5.57508 | -1.851 | 0.03553 | NA | NA |
| cfa-miR-485 | 13.8391 | 79.1649 | -1.8607 | 0.01589 | 0.06927 | FALSE |
| cfa-miR-486 | 211731 | 6176.34 | 3.6886 | NA | NA | NA |
| cfa-miR-486-3p | 207437 | 6080.89 | 3.6785 | NA | NA | NA |
| cfa-miR-487a | 7.10566 | 5.65903 | 0.20428 | 0.81467 | NA | NA |
| cfa-miR-487b | 16.3908 | 61.5405 | -1.3844 | 0.07726 | 0.20793 | FALSE |
| cfa-miR-488 | 3.31947 | 2.90897 | 0.07881 | 0.92821 | NA | NA |
| cfa-miR-489 | 0.16735 | 0.88479 | -0.3835 | 0.56397 | NA | NA |
| cfa-miR-490 | 103.892 | 117.821 | -0.1595 | 0.77157 | 0.87606 | FALSE |
| cfa-miR-491 | 6.74887 | 2.56906 | 0.95355 | 0.23462 | NA | NA |
| cfa-miR-493 | 33.6083 | 125.736 | -1.4954 | 0.03814 | 0.13086 | FALSE |
| cfa-miR-494 | 83.6249 | 184.753 | -0.9187 | 0.18735 | 0.35826 | FALSE |
| cfa-miR-495 | 116.457 | 209.333 | -0.6885 | 0.31148 | 0.51834 | FALSE |
| cfa-miR-497 | 2946.02 | 3988.46 | -0.4018 | 0.40211 | 0.63522 | FALSE |
| cfa-miR-499 | 56169.5 | 29324.7 | 0.85749 | 0.08093 | 0.21257 | FALSE |
| cfa-miR-500 | 16441 | 14619.2 | 0.15572 | 0.74559 | 0.85999 | FALSE |
| cfa-miR-502 | 22109.1 | 18453.2 | 0.2403 | 0.61159 | 0.78427 | FALSE |
| cfa-miR-503 | 170.502 | 177.965 | -0.0578 | 0.91257 | 0.96106 | FALSE |
| cfa-miR-504 | 349.534 | 1136.75 | -1.6138 | 3.21E-05 | 0.0014 | TRUE |
| cfa-miR-505 | 199.146 | 138.757 | 0.49468 | 0.18149 | 0.35012 | FALSE |
| cfa-miR-506 | 73.6064 | 1.07235 | 3.146 | NA | NA | NA |
| cfa-miR-507a | 270.371 | 8.85309 | 2.9132 | NA | NA | NA |
| cfa-miR-507b | 13.7024 | 0.94527 | 1.3903 | NA | NA | NA |
| cfa-miR-508a | 47.5139 | 2.93289 | 2.1664 | NA | NA | NA |
| cfa-miR-508b | 278.433 | 6.55087 | 3.3427 | NA | NA | NA |
| cfa-miR-514 | 17.7215 | 0 | 2.5094 | NA | NA | NA |
| cfa-miR-532 | 183618 | 71078.8 | 1.1901 | NA | NA | NA |
| cfa-miR-539 | 1.2206 | 10.899 | -1.5397 | NA | NA | NA |
| cfa-miR-542 | 933.628 | 1239.7 | -0.3712 | 0.46666 | 0.6908 | FALSE |
| cfa-miR-543 | 64.0522 | 83.1852 | -0.3001 | 0.65835 | 0.82012 | FALSE |
| cfa-miR-551a | 627.135 | 304.64 | 0.8627 | 0.19175 | 0.36348 | FALSE |
| cfa-miR-551b | 0.27559 | 0 | 0.12677 | 0.74125 | NA | NA |
| cfa-miR-574 | 2157.45 | 6579.04 | -1.5236 | 0.00011 | 0.0031 | TRUE |
| cfa-miR-582 | 40.6967 | 63.4335 | -0.4981 | 0.49153 | 0.69891 | FALSE |
| cfa-miR-590 | 4500.9 | 4350.78 | 0.04111 | 0.9493 | 0.9767 | FALSE |
| cfa-miR-592 | 122.117 | 257.274 | -0.9457 | 0.0965 | 0.24181 | FALSE |
| cfa-miR-599 | 0.33471 | 0.63018 | -0.1088 | 0.84971 | NA | NA |
| cfa-miR-628 | 207.178 | 317.497 | -0.5841 | 0.1393 | 0.29483 | FALSE |
| cfa-miR-632 | 93.7762 | 146.425 | -0.5852 | 0.24112 | 0.42391 | FALSE |
| cfa-miR-6516 | 79.2934 | 68.0801 | 0.19405 | NA | NA | NA |
| cfa-miR-652 | 2167.85 | 1883.98 | 0.195 | 0.55341 | 0.74472 | FALSE |
| cfa-miR-6529 | 466.652 | 130.291 | 1.482 | NA | NA | NA |
| cfa-miR-653 | 0.66942 | 1.46622 | -0.3015 | 0.70818 | NA | NA |
| cfa-miR-660 | 6006.34 | 4762.65 | 0.31297 | 0.47079 | 0.6908 | FALSE |
| cfa-miR-664 | 60.1855 | 17.0447 | 1.6097 | 0.00533 | 0.03745 | TRUE |
| cfa-miR-671 | 2363.82 | 643.516 | 1.542 | NA | NA | NA |
| cfa-miR-676 | 13.4056 | 21.8237 | -0.53 | 0.47849 | 0.6908 | FALSE |
| cfa-miR-7 | 1008863 | 314631 | 1.4847 | 0.00829 | 0.05013 | FALSE |
| cfa-miR-708 | 1270.99 | 1664.98 | -0.3564 | 0.46689 | 0.6908 | FALSE |
| cfa-miR-7180 | 2298.2 | 1070.44 | 0.95634 | 0.10755 | 0.25764 | FALSE |
| cfa-miR-758 | 10.5505 | 28.4711 | -1.0914 | 0.13573 | 0.29057 | FALSE |
| cfa-miR-769 | 206.959 | 108.844 | 0.85902 | 0.05511 | 0.1692 | FALSE |
| cfa-miR-874 | 475.842 | 446.526 | 0.08106 | 0.87811 | 0.93837 | FALSE |
| cfa-miR-875 | 3.28798 | 0 | 0.98002 | NA | NA | NA |
| cfa-miR-876 | 0 | 1.18162 | -0.466 | NA | NA | NA |
| cfa-miR-8800 | 0.92801 | 0 | 0.47973 | 0.46612 | NA | NA |
| cfa-miR-8803 | 112.284 | 16.0746 | 2.0268 | NA | NA | NA |
| cfa-miR-8809 | 0.55118 | 0 | 0.22676 | 0.64655 | NA | NA |
| cfa-miR-8810 | 0 | 0.31509 | -0.1499 | 0.66561 | NA | NA |
| cfa-miR-8813 | 0.29269 | 0 | 0.13349 | 0.73407 | NA | NA |
| cfa-miR-8820 | 0.33471 | 0.31509 | 0.01169 | 0.98218 | NA | NA |
| cfa-miR-8824 | 0.58538 | 0 | 0.23755 | 0.63749 | NA | NA |
| cfa-miR-8826 | 3.71209 | 1.41792 | 0.47872 | 0.57439 | NA | NA |
| cfa-miR-8829 | 146.626 | 29.6493 | 2.0623 | 0.0001 | 0.0031 | TRUE |
| cfa-miR-8835 | 0.55118 | 0 | 0.22676 | 0.64655 | NA | NA |
| cfa-miR-8842 | 2.40131 | 6.37456 | -0.8857 | 0.30177 | NA | NA |
| cfa-miR-885 | 25.8473 | 69.8547 | -1.3198 | 0.00422 | 0.03338 | TRUE |
| cfa-miR-8858 | 48.5895 | 86.6342 | -0.7739 | 0.08435 | 0.21634 | FALSE |
| cfa-miR-8859a | 817.01 | 999.163 | -0.2738 | 0.49961 | 0.70267 | FALSE |
| cfa-miR-8859b | 475.734 | 406.896 | 0.20671 | 0.66444 | 0.823 | FALSE |
| cfa-miR-8865 | 149.733 | 47.2843 | 1.4401 | 0.01532 | 0.06815 | FALSE |
| cfa-miR-8871 | 0.27559 | 0.94527 | -0.2441 | 0.68617 | NA | NA |
| cfa-miR-8872 | 11.4961 | 9.55036 | 0.21539 | 0.76737 | NA | NA |
| cfa-miR-8876 | 13.5429 | 4.21155 | 1.1239 | 0.16379 | NA | NA |
| cfa-miR-8883 | 1.86731 | 0 | 0.51923 | 0.44143 | NA | NA |
| cfa-miR-8884 | 17705.7 | 9780.95 | 0.77686 | 0.12721 | 0.28716 | FALSE |
| cfa-miR-889 | 245.563 | 745.105 | -1.3025 | 0.0584 | 0.17205 | FALSE |
| cfa-miR-8890 | 4.72981 | 1.1394 | 0.69394 | 0.41641 | NA | NA |
| cfa-miR-8898 | 0.46683 | 0 | 0.19671 | 0.67278 | NA | NA |
| cfa-miR-8902 | 0 | 1.51453 | -0.5535 | 0.35976 | NA | NA |
| cfa-miR-8903 | 17.7092 | 7.3616 | 1.037 | 0.12988 | NA | NA |
| cfa-miR-8908a-3p | 30.5572 | 462.18 | -1.6687 | NA | NA | NA |
| cfa-miR-8908a-5p | 1.2378 | 87.9333 | -3.6241 | NA | NA | NA |
| cfa-miR-8908b | 2.12572 | 157.771 | -3.8442 | NA | NA | NA |
| cfa-miR-8908c | 2.25284 | 10.4146 | -1.0562 | NA | NA | NA |
| cfa-miR-8908d | 14.9973 | 104.847 | -1.0989 | 0.20099 | 0.37131 | FALSE |
| cfa-miR-8908f | 0 | 1.45449 | -0.7841 | 0.2535 | NA | NA |
| cfa-miR-9 | 28439.3 | 3534.18 | 2.2448 | NA | NA | NA |
| cfa-miR-92a | 286288 | 73652.8 | 1.7467 | 0.00131 | 0.0159 | TRUE |
| cfa-miR-92b | 49.8881 | 9.8397 | 1.8667 | 0.00772 | 0.04809 | TRUE |
| cfa-miR-93 | 52281.2 | 21352 | 1.1885 | 0.01258 | 0.0662 | FALSE |
| cfa-miR-95 | 408.915 | 671.423 | -0.6561 | 0.17206 | 0.33792 | FALSE |
| cfa-miR-96 | 12280.5 | 1586.53 | 2.3668 | NA | NA | NA |
| cfa-miR-98 | 24950.2 | 23535.8 | 0.08017 | 0.83108 | 0.91502 | FALSE |
| cfa-miR-99a | 2639476 | 2815408 | -0.0784 | 0.90281 | 0.95641 | FALSE |
| cfa-miR-99b | 46587.7 | 33032.7 | 0.43929 | 0.4326 | 0.66884 | FALSE |

**Supplementary Table 7:** The exported calibrated Cq for all samples.

| **miRNA ID** | **Samples ID** | | | | | | | | | |
| --- | --- | --- | --- | --- | --- | --- | --- | --- | --- | --- |
|  | **Healthy_3** | **Healthy_4** | **Healthy_5** | **Healthy_6** | **DLBCL 1** | **DLBCL 2** | **DLBCL 3** | **DLBCL 4** | **DLBCL 5** | **DLBCL 6** |
| UniSp6 | 21.509 | 20.501 | 21.027 | 20.804 | 21.564 | 20.826 | 21.146 | 20.3 | 21.119 | 20.331 |
| UniSP3 | 21.638 | 21.639 | 21.637 | 21.638 | 21.638 | 21.639 | 21.638 | 21.638 | 21.638 | 21.638 |
| U6 snRNA | 23.17 | 23.361 | 21.799 | 25.919 | 21.784 | 24.668 | 24.364 | 24.414 | 20.46 | 23.407 |
| has-miR-217 | 29.263 | 30.723 | 29.746 | 30.379 | 35.723 | 36.917 | 38.013 | 31.83 | 36.874 | 36.759 |
| cfa-miR-132 | 33.314 | 34.557 | 34.192 | 33.956 | 36.873 | 34.476 | 35.051 | 36.223 | 35.985 | 34.847 |
| hsa_miR_378a-3p | 27.603 | 28.699 | 27.923 | 27.93 | 27.434 | 29.31 | 26.361 | 27.601 | 28.92 | 26.768 |
| hsa_miR_192-5P | 29.69 | 30.145 | 29.563 | 29.432 | 29.575 | 30.301 | 28.494 | 29.177 | 29.158 | 28.551 |
| hsa_miR_20a_5p | 25.286 | 25.294 | 25.145 | 24.763 | 23.725 | 23.646 | 22.875 | 23.972 | 23.879 | 23.111 |
| hsa_miR_128_3p | 28.487 | 28.771 | 28.413 | 29.816 | 28.53 | 29.182 | 28.128 | 30.046 | 28.009 | 28.916 |
| cfa_miR_106a | 25.403 | 25.672 | 25.659 | 25.582 | 24.402 | 23.905 | 23.428 | 24.549 | 24.259 | 23.892 |
| hsa_miR_92a_3p | 23.915 | 24.286 | 23.595 | 24.691 | 22.958 | 22.989 | 22.388 | 23.59 | 23.034 | 23.64 |
| cfa_miR_1842 | 32.187 | 31.682 | 31.108 | 33.11 | 31.531 | 30.977 | 30.142 | 31.926 | 31.765 | 30.641 |
| bta_miR_20b | 28.666 | 29.887 | 29.227 | 28.977 | 27.708 | 26.925 | 25.919 | 28.108 | 29.18 | 27.137 |
| hsa_miR_29c_3p | 23.972 | 23.886 | 23.634 | 24.288 | 23.836 | 24.629 | 22.986 | 23.847 | 23.612 | 23.899 |
| hsa_miR_425-5p | 26.402 | 28.283 | 26.888 | 26.931 | 26.25 | 26.939 | 26.026 | 28.226 | 26.617 | 26.492 |
| cfa_miR_19b | 23.32 | 23.608 | 23.567 | 22.924 | 22.078 | 22.7 | 22.855 | 22.151 | 22.996 | 21.912 |
| cfa_miR_144 | 35.444 | 35.208 | 35.543 | 34.909 | 32.673 | 31.952 | 35.811 | 33.489 | 33.994 | 32.501 |
| cfa_miR_1840 | 37.872 | 36.585 | 35.74 | 37.661 | 36.651 | 36.588 | 35.851 | 36.74 | 37.99 | 36.021 |
| gga_miR_18a_5p | 28.98 | 30.151 | 29.807 | 29.801 | 28.193 | 28.395 | 28.168 | 28.678 | 28.719 | 28.176 |
| hsa_miR_17-3p | 30.827 | 30.92 | 30.065 | 30.697 | 28.464 | 28.97 | 29.321 | 29.13 | 28.544 | 29.13 |
| cfa_mir_664 | 38.494 | 38.239 | 37.7 | 38.777 | 37.029 | 37.186 | 36.439 | 37.609 | 37.272 | 35.569 |
| hsa_miR_92b_3p | 38.652 | 38.488 | 39.333 | 38.881 | 38.8385062 | 39.433 | 36.724 | 38.332018 | 37.886 | 39.907 |
| hsa_miR_150_5p | 22.475 | 22.319 | 22.612 | 22.786 | 26.489 | 25.236 | 23.987 | 25.893 | 25.113 | 25.684 |
| hsa_miR_504_5p | 37.097 | 37.729 | 36.215 | 38.716 | 38.984 | 38.794 | 38.849068 | 39.417 | 38.201 | 39.034 |
| hsa_miR_574_3p | 31.274 | 32.327 | 30.077 | 32.751 | 35.051 | 31.99 | 34.622 | 33.996 | 33.344 | 33.782 |
| hsa_miR_146a_5p | 24.792 | 25.527 | 25.188 | 25.152 | 28.317 | 27.359 | 28.191 | 28.238 | 26.993 | 28.89 |
| hsa_miR_151a_5p | 26.388 | 27.028 | 26.696 | 26.45 | 28.278 | 27.828 | 30.525 | 28.403 | 28.46 | 28.107 |
| hsa_miR_216b_5p | 31.558 | 30.467 | 29.913 | 30.387 | 33.832 | 35.555 | 35.544 | 30.813 | 33.922 | 33.826 |
| hsa_miR379_5p | 31.603 | 33.544 | 33.413 | 32.765 | 36.586 | 33.636 | 38.777 | 35.4 | 38.153 | 34.199 |
| hsa_miR_885_5P | 32.888 | 34.431 | 33.125 | 34.183 | 35.844 | 35.736 | 36.973 | 35.105 | 37.333 | 35.533 |
| hsa_miR_152_3P | 27.854 | 28.972 | 28.766 | 28.456 | 31.663 | 28.989 | 30.549 | 29.928 | 30.735 | 29.607 |
| cfa_miR_139 | 36.118 | 35.144 | 31.835 | 35.573 | 36.525 | 36.23 | 35.679 | 37.439 | 32.417 | 33.442 |
| hsa_miR_21_5p | 20.969 | 22.087 | 21.29 | 21.17 | 23.094 | 22.046 | 22.789 | 23.659 | 23.138 | 21.934 |
| cfa_miR_101 | 26.879 | 26.492 | 26.113 | 25.61 | 27.082 | 26.58 | 26.838 | 26.896 | 27.244 | 26.339 |
| hsa_miR_22_3p | 25.737 | 26.185 | 25.875 | 26.215 | 27.445 | 26.738 | 28.222 | 27.937 | 26.136 | 26.835 |
| bta_miR99a_5p | 25.089 | 25.823 | 25.445 | 25.165 | 25.999 | 26.783 | 30.076 | 26.677 | 25.88 | 27.277 |
| hsa_miR_98_5P | 27.452 | 28.12 | 27.889 | 27.762 | 28.888 | 28.571 | 27.717 | 28.643 | 28.879 | 28.64 |
| hsa_miR_218_5p | 30.203 | 32.061 | 31.162 | 30.938 | 32.829 | 30.999 | 33.939 | 32.766 | 31.937 | 31.369 |
| hsa_miR_129_5p | 36.369 | 37.015 | 36.326 | 35.911 | 37.703 | 38.809 | 36.15 | 39.457 | 38.707 | 39.097 |
| cfa_miR_15a | 26.415 | 26 | 25.88 | 26.424 | 27.479 | 26.588 | 25.767 | 26.944 | 26.826 | 26.488 |
| cfa_miR_450a | 32.656 | 32.745 | 32.637 | 31.871 | 35.915 | 33.3 | 32.523 | 31.68 | 35.417 | 33.772 |
| hsa_miR_361_5p | 27.55 | 27.668 | 27.506 | 27.991 | 28.86 | 28.34 | 27.76 | 28.312 | 28.916 | 28.307 |
| hsa_miR_451a | 26.703 | 27.393 | 28.235 | 28.08 | 23.686 | 23.382 | 24.006 | 26.291 | 23.913 | 24.894 |
| cfa_miR_31 | 29.142 | 29.517 | 29.733 | 29.189 | 26.069 | 25.915 | 25.558 | 25.234 | 29.074 | 24.537 |
| hsa_miR_34a_5p | 29.729 | 30.158 | 28.762 | 30.646 | 26.351 | 26.982 | 30.95 | 26.618 | 27.315 | 26.294 |
| cfa_miR_363 | 27.479 | 28.202 | 27.146 | 27.499 | 26.999 | 25.444 | 25.14 | 27.06 | 29.089 | 26.301 |
| mmu_miR_1839 | 31.868 | 33.134 | 32.131 | 32.554 | 30.853 | 31.248 | 30.449 | 30.986 | 33.766 | 30.744 |
